# TFEB and TFE3 cooperate in regulating inorganic arsenic-induced autophagy-lysosome impairment and immuno-dysfunction in primary dendritic cells

**DOI:** 10.1007/s10565-024-09841-0

**Published:** 2024-01-25

**Authors:** Guowei Xu, Huaguang Peng, Ran Yao, Yuqing Yang, Bing Li

**Affiliations:** 1 Key Laboratory of Environmental Stress and Chronic Disease Control and Prevention, Ministry of Education (China Medical University), Shenyang, Liaoning People’s Republic of China; 2 Environment and Non-Communicable Disease Research Center, Key Laboratory of Arsenic-Related Biological Effects and Prevention and Treatment in Liaoning Province, School of Public Health, China Medical University, No.77 Puhe Road, Shenyang North New Area Liaoning Province, Shenyang, 110122 People’s Republic of China

**Keywords:** Inorganic arsenic, Dendritic cells, Autophagy, Lysosome, TFEB, TFE3

## Abstract

Arsenic (As) is a prevalent and hazardous environmental toxicant associated with cancer and various health problems, which has been shown suppressive effects on dendritic cells (DCs). Autophagy is essential for the innate and adaptive immune responses of DCs, and the transcription factors TFEB and TFE3 are key regulators of autophagic and lysosomal target genes. However, the detrimental alterations of the autophagy-lysosome pathway in As-exposed DCs and the possible coordinating roles of TFEB and TFE3 in the immune dysfunction of this cell are less understood. In this paper, we found that As exposure significantly impaired lysosomal number, lysosomal acidic environment, and lysosomal membrane permeabilization, which might lead to blocked autophagic flux in cultured DCs. Furthermore, our results confirmed that TFEB or TFE3 knockdown exacerbated the disorders of lysosome and the blockade of autophagic flux in As-exposed DCs, and also enhanced the inhibitory expression of co-stimulatory molecules *Cd80* and *Cd83*; adhesion molecule *Icam1*; cytokines TNF-α, IL-1β, and IL-6; chemokine receptor *Ccr7*; and antigen-presenting molecules MHC II and MHC I. By contrast, overexpression of TFEB or TFE3 partially alleviated the above-mentioned impairment of DCs by inorganic As exposure. In conclusion, these findings reveal a previously unappreciated inhibition of lysosome-mediated degradation and damage of lysosomal membrane integrity leading to dysregulated autophagy and impaired immune functions of DCs by arsenicals, and also suggest TFEB and TFE3 as potential therapeutic targets for ameliorating As toxicity.

**Graphical abstract:**

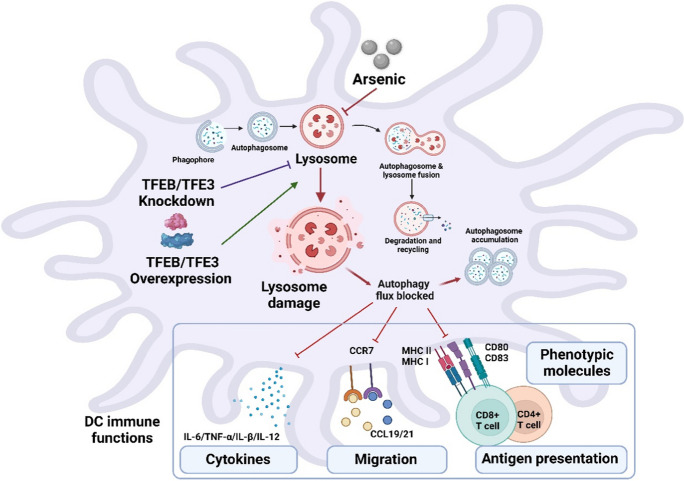

**Supplementary Information:**

The online version contains supplementary material available at 10.1007/s10565-024-09841-0.

## Introduction

Arsenic (As) is one of the most prevalent environmental toxicants and ranks first on the ATSDR’s Priority List of Hazardous Substances due to its toxicity and potential for human exposure (https://wwwn.cdc.gov/TSP/substances/ToxSubstance.aspx?toxid=3). As a known group I human carcinogen, it is considered to be a major causative factor in some human cancers, such as skin, lung, and bladder cancer. It is also suggested to cause a widespread range of acute and chronic health effects, including cardiovascular, neurological and renal disorders, and diabetes (Farkhondeh et al. 2019; Garza-Lombo et al. 2019; Kaur et al. 2022; Zhang et al. 2019). What’s more, studies now believe that diseases mentioned above may be related to aberrant immune function in the human body, making the immunotoxicity of As one of the hot research topics.

Dysregulated immunity may contribute to the progression of As-induced cancers (Huang et al. 2019). Selective CD4 + T cell apoptosis, reduced macrophage differentiation and phagocytosis, decreased number of Langerhans cell and dendrites, and altered distribution of regulatory T cells have been identified in patients with As-induced Bowen’s disease (Lee et al. 2011). Furthermore, both CD4 + intraepithelial lymphocytes (TCRαβ + and CD8αα +) and the innate immune macrophages in the lamina propria of the small intestinal are sensitive to As and reduced after chronic drinking water exposure of mice to sodium arsenite (Medina et al. 2020). As one of the critical immune cells, dendritic cells (DCs) are the link between innate immunity and adaptive immunity, playing important roles in both promoting immune defense and maintaining immune tolerance (Liu et al. 2021). Arsenicals have recently been reported to have suppressive effects on DCs, inducing abnormal immune responses and thereby increasing susceptibility to a variety of diseases. Treatment with arsenic trioxide (ATO) was shown to reduce the expression of major histocompatibility complex II (MHC II), CD80, and CD86 in the membrane of DCs, which are involved in antigen presentation and co-stimulatory signals to activate T cells (Ye et al. 2020). Our previous results also reported that inorganic arsenic downregulates the expression of phenotypic molecules and cytokines and thus significantly induces immune tolerance in LPS-stimulated BMDCs (Li et al. 2021). However, the specific mechanisms involved are still not very clear.

As a well-known lysosome-mediated degradation pathway providing self-protection to the organism (Kim and Lee 2014), developments in the last decade also suggest autophagy as one of the key mechanisms orchestrating the differentiation and metabolic state of immune cells. In particular, several reports highlight the indirect or direct involvement of autophagy in the innate and especially adaptive immune responses of DCs, including DC maturation, cytokine production, migration, antigen presentation, and activation of T cell (Ghislat and Lawrence 2018). On the other hand, autophagy relies mainly on lysosome for the final degradation of intracellular components, and lysosome is nowadays considered as a novel central and dynamic organelle in cell homeostasis, metabolic regulation, and immune system, including endocytosis, phagocytosis, antigen processing, secretory mechanisms, and cell death pathways (Vargas et al. 2023; Yim and Mizushima 2020). Trivalent As has been reported to induce autophagy-related protein (LC3-II, Atg-5, Beclin-1) expression, autophagosome accumulation, and autophagy-dependent apoptosis in a few cell lines (Fu et al. 2021; Liang et al. 2020), while also downregulates autophagic flux in human kidney cells, lung epithelial cells, and keratinocytes, leading to autophagosome accumulation and sequestration of p62, Keap1, and LC3-II (Lau et al. 2013; Wu et al. 2019). In our published work, we observed a decrease in the number of lysosomes by transmission electron microscopy and attenuated protein expression of lysosomal-specific indicators LAMP1, LAMP2, and lysosomal cysteine cathepsins CTSL and CTSD in As-treated BMDCs (Zhao et al. 2020). However, the more detailed effects of inorganic As on the impaired lysosomal degradation functions, including lysosomal acidic environment, proton pump expression, and lysosomal membrane integrity, remain to be elucidated, as these lysosomal degradative functions are essential and critical for the outcomes of intracellular components including autophagosome, and the autophagy-lysosome pathway is essential for maintaining normal immune functions in DCs. What’s more, these lysosomal injuries have not been previously reported.

It is well established that the MiT/TFE transcription factor TFEB is key manager of the autophagy-lysosomal pathway by regulating the expression of multiple autophagic and lysosomal target genes, such as lysosomal membrane proteins, lysosomal hydrolases, and subunits of the v-ATPase by binding to CLEAR elements in the promoters of these genes (Contreras et al. 2023; Sardiello et al. 2009). Meanwhile, TFEB has been reported to regulate exogenous antigen presentation and DC migration through lysosomal signaling (Samie and Cresswell 2015; Vestre et al. 2021). In addition to TFEB, the roles of another MiT family member TFE3 have attracted increasing attention in recent years. Overexpression of either TFEB or TFE3 is sufficient to increase the levels of numerous chemokines and cytokines in cellular response to virus infection (Campbell et al. 2015; Contreras et al. 2023). Previously, we showed that overexpression of TFEB by lentiviral transfection partially rescued As-inhibited lysosomal CTSD and CTSL expressions, improved the disorder of autophagic flux, and affected immunocytokine expression in cultured BMDCs (Zhao et al. 2020). Nevertheless, the possible involvement and the coordinating characters of TFEB and TFE3 in modulating the essential lysosomal degradative functions, as well as the impaired immune functions (e.g., antigen phagocytosis, antigen degradation, and antigen presentation) in As-exposed DCs, are less understood yet.

In the present study, we described that impairment of DC lysosomal degradative function by As exposure was a possible mechanism by which As blocked autophagic flux and consequently led to abnormal functions in DCs. Furthermore, as key regulatory transcription factors of autophagy-lysosome pathway (ALP), knockdown of TFEB or TFE3 exacerbated the reduction of lysosomal number, disruption of lysosomal acidic environment, increase of lysosomal membrane permeabilization (LMP), and obstruction of the autophagic flux in As-exposed DCs. It also enhanced the inhibitory effects of As on phenotypic molecules and cytokine expression, migration, and antigen-presenting function of DCs. By contrast, overexpression of TFEB or TFE3 may contribute to lysosomal biogenesis and promote the expression of autophagy-related genes in DCs, thereby activating ALP and providing some protection against impaired immune function due to As exposure.

## Materials and methods

### Cell culture and treatment

Primary cultures of BMDCs were prepared from the bone marrow of C57BL/6 mice with the approval by the Institutional Animal Care and Use Committees of China Medical University. Briefly, the bone marrow was separated and collected from the femurs and tibias of C57BL/6 mice under sterile conditions. After removal of red blood cells, the bone marrow cells were washed with PBS (Thermo Fisher Scientific, hereafter as Thermo, 20,012,027) and filtered through a 70-μm cell strainer. The bone marrow cells were then plated in a 6-well plate and cultured in fresh RPMI 1640 (Thermo, 11,875,119) growth medium supplemented with 10% FBS (Thermo, 10100147C), 100 U/mL penicillin/streptomycin (Thermo, 15,070,063), L-glutamine (20 mM, Thermo, A2916801), 1% HEPES buffer solution (Thermo, 15,630,130), murine IL-4 (5 ng/mL, PeproTech, 214–14), and GM-CSF (5 ng/mL, PeproTech, 315–03). Half of the culture medium was replaced after 12 h and the medium was then refreshed every other day. BMDCs were finally obtained by gently harvesting the semi-adherent cells from the culture dishes on day 7. Over 80% of the cell population was positive for the dendritic cell-specific marker CD11c (Thermo, 12–0114-82), as confirmed by flow cytometry (BD Biosciences, USA, CantoII) (Fig. S1).

The following treatments were added to the cells: sodium arsenite (1 μM, 6, 12, and 24 h, Sigma-Aldrich, hereafter as Sigma, S7400), lipopolysaccharide (25 ng/mL, 6 and 12 h, Sigma, SMB00704), rapamycin (200 nM, Sigma, V900930), 3-methyladenine (3-MA, 1 mM, Sigma, M9281), and chloroquine (CQ, 20 μM, GlpBio, GC19549). Rapamycin, 3-MA, and CQ pre-treated BMDCs for 1 h and then co-treated with arsenic for 12 h. Treated cells were harvested at various times for the subsequent experiments.

### Immunolabeling by flow cytometry

The different groups BMDCs were initially pre-treated with PBS containing 5% FBS for 20 min to obviate non-specific binding. Afterward, the flow cytometry was assessed as previously described (Li et al. 2021). BMDCs were separately incubated with appropriate anti-CD11c (PE-labeled, Thermo, 12–0114-82), anti-MHC II (FITC-labeled, Thermo, 11–5321-82), and anti-MHC I (PE Cyanine7-labeled, Thermo, 25–5999-82) for 30 min at 4 ℃. Blank and isotype controls were set concurrently. Finally, BMDCs were washed and immunolabeled fluorescence was measured using a flow cytometer (BD Biosciences, USA, LSRFortessa) at a rate of 10,000 events per sample.

### Western blotting analysis

To extract whole-cell proteins, BMDCs were lysed in RIPA Cell Lysis Buffer (Sigma, V900854) containing protease and phosphatase cocktail inhibitor (Sigma, PPC1010). The lysates were sonicated and then centrifuged at 12,000 g for 15 min at 4 °C to collect whole-cell proteins in the supernatant. The lysosomal fraction was isolated using the Lysosome Isolation Kit (Sigma, LYSISO1) according to the manufacturer’s instructions.

Protein concentrations were measured using the BCA Protein Assay Kit (Thermo, 23,225). Thirty micrograms of protein per sample was separated by 10% SDS-PAGE and transferred to 0.2-μm PVDF membranes (Millipore, ISEQ00010). Membranes were blocked with 5% BSA (Sigma, V900933) or 5% non-fat milk for 1 h and then incubated with primary antibodies overnight at 4 °C. On the second day of the experiment, the membranes were washed and then exposed to the respective secondary antibodies diluted 1:2000–5000 for 2 h at room temperature. Blots were covered with the ECL (Tanon, 180–506) and visualized using an Electrophoresis Gel Imaging Analysis System (Azure Biosystems, USA, C500).

For the whole-cell protein samples, band densities were normalized to β-actin as a loading control. For the lysosomal fraction, LAMP2 was used as a loading control. Details of the primary antibodies used for western blotting are shown in Table S1.

### RNA isolation and real-time PCR (RT-PCR)

The Trizol method was used to extract total RNA from BMDCs. The PrimeScript^TM^RT reagent Kit (Takara, RR047A) was used for reverse transcription of the RNA into cDNA using a gradient PCR instrument (IMPLEN, Germany, N60 Touch). The TB Green ® Premix Ex Taq™ fluorescence quantification kit (Takara, RR820A) was used to prepare the reaction solution, and the reaction was performed using a two-step PCR amplification procedure with a QuantStudio 6 Flex Real-Time PCR System (ABI, USA). The relative differences in gene expression were calculated using the cycle threshold (Ct) values according to Eq. 2^−△△Ct^. Primers for the corresponding genes were designed and synthesized by Sangon Biotech (Shanghai, China), which are listed in Table S2.

### Lysosomal staining

Fluorescent probes Lyso-Tracker (Beyotime, C1046) and Lyso-Sensor (Yeasen, 40767ES50) were used to label lysosome in primary BMDCs. Lyso-Tracker exhibits pH-independent fluorescence, while Lyso-Sensor exhibits a pH-dependent fluorescence. Therefore, we used Lyso-Tracker to detect lysosomal abundance and Lyso-Sensor to assess the relative changes in lysosomal pH. BMDCs were cultured on 14-mm glass bottom dishes coated with poly-d-lysine. After exposure to arsenic or other treatments, BMDCs were incubated with 50 nM Lyso-Tracker Red or 1 μM Lyso-Sensor Green in culture medium for 30 min at 37 °C, and then cells were gently rinsed twice with warm PBS. BMDCs visualized live using a Nikon Ni-U fluorescence microscope. Immunofluorescence images were captured from randomly selected fields to evaluate the experimental outcomes.

### Immunofluorescence assay

After the indicated treatments, BMDCs were harvested and fixed with 4% paraformaldehyde (Sigma, 818,715) for 15 min and permeabilized with 0.5% Triton X-100 (Thermo, 85,111) for 20 min. Next, cells were incubated with BlockAid™ blocking solution (Thermo, B10710) for 1 h at room temperature, followed by overnight incubation at 4 °C with primary antibodies, which are listed in Table S3. Subsequently, cells were further incubated with the appropriate secondary antibodies for 1 h. Nuclei were stained with DAPI (Thermo, S36939) and samples were imaged by fluorescence microscopy (Nikon Ni-U).

### Plasmid construction

The CRISPR/Cas9 knockdown technique was utilized to generate the TFEB/TFE3 knockdown plasmid (Plasmid-TFEB/TFE3 KD) and the negative control plasmid (NC, Plasmid-TFEB/TFE3 scrambled). Mouse *Tfeb* and *Tfe3* sequences were obtained and target sequences were designed according to sgRNA target design principles, with nonsense sequences serving as sgRNA negative controls. The targeting sequences used are shown in Table S4. These target and nonsense sequences were cloned into the backbone vector (plasmid vector information: pLV-hU6-sgRNA-EFS-hCas9-2A-Puro) to generate the target plasmid. The gene editing plasmid was synthesized, transformed, and extracted, and the target gene sequence was sequenced at the target site and compared to identify successful plasmid construction for subsequent experiments. Furthermore, using the exogenous gene overexpression technique, the TFEB/TFE3 overexpression plasmid (Plasmid-TFEB/TFE3 OE) and negative control plasmid (NC, Plasmid-TFEB/TFE3 scrambled) were constructed. The *Tfeb*, *Tfe3*, and the nonsense sequences were designed and cloned into the plasmid vector (plasmid vector information: ppCDNA3.1( +)-CMV-MCS-3Flag) to generate the overexpression plasmids. The subsequent steps of plasmid transformation, extraction, and sequencing were carried out as described above.

### Cell transfection

For the generation of a transient cell model of TFEB/TFE3 knockdown or overexpression in mouse primary BMDCs, electrotransfection was utilized. Firstly, the electrotransfection solution was prepared by mixing Nucleofector Solution from the Lonza transfection kit (Lonza, V4XP-3012) with supplement in a 4.5:1 ratio. The collected BMDCs were then resuspended in the prepared electrotransfection solution and transferred to the electrotransfection cups. Subsequently, the TFEB/TFE3 knockdown plasmid (Plasmid-TFEB/TFE3 KD) generated by CRISPR/Cas9 knockdown technique, TFEB/TFE3 overexpression plasmid constructed by exogenous overexpression technique (Plasmid-TFEB/TFE3 OE) or the negative control plasmid (NC, Plasmid-TFEB/TFE3 scrambled) was separately added to the electrotransfection cups according to the experimental design. After gently mixed, the electrode cups were then placed in the X-unit of the electrotransfer instrument, and the Nucleofector System (Lonza Group, Swiss) was initiated with the DJ-100 program selected for immature dendritic cells and the DN-107 program selected for mature dendritic cells (https://knowledge.lonza.com/). After completion of the electrotransfection process, the electrode cups were removed and the BMDCs were resuspended in pre-warmed RPMI-1640 culture medium. The transfection efficiency was evaluated by observing the fluorescence of the cells after 4–24 h, and subsequent experiments were carried out accordingly.

### ELISA

The supernatants obtained from the BMDCs were collected, and ELISA kits were used to assess the levels of tumor necrosis factor (TNF)-α (Thermo, 88–7324), interleukin (IL)-1β (Thermo, 88–7013), and IL-6 (Thermo, 88–7064) following the manufacturer’s instructions. The absorbance of the samples was measured at 450 nm using a microplate reader (Biotech, H1MD). Cytokine concentrations were calculated and expressed in pg/mL.

### Transwell migration assay

The experimental setup consisted of 200 μL BMDCs in an upper chamber with an 8-μm pore size Transwell, followed by 600 μL 1640 medium containing CCL19 (50 ng/mL, PeproTech, 250-27B) and CCL21 (50 ng/mL, PeproTech, 250–13) in the lower chamber. The upper chamber was then nested into the lower chamber and incubated in a CO_2_ incubator for 4 h. Subsequently, the upper chamber was removed and the BMDCs in the lower chamber were counted using a cell counter (ALIT Life science, China, IC1000). Migration rate (%) was finally calculated as the ratio of the cell numbers in the lower chamber to those in the upper chamber.

### Antigen cross-presentation assay

The cross-presentation of ovalbumin (OVA) antigen (Sigma, A5503) by DCs to B3Z CD8 + T hybridoma cells was assessed using β-galactosidase (lacZ) color conversion. BMDCs were cultured with 1 mg/mL OVA for 24 h. OVA-pulsed BMDCs were then co-cultured with B3Z CD8 + T hybridoma cells in a 96-well U-bottom plate for a further 24 h. The centrifugated cells were then added to 150 μL CPRG lysis buffer (PBS supplemented with 0.15 mM chlorophenol red-β-galactopyranoside [GlpBio, GC47080] and [0.125% NP40 GlpBio, GF05305]) for 24 h under light avoidance in a CO_2_ incubator. Finally, 100 μL of the supernatant was transferred to a new 96-well flat-bottom plate, and the absorbance of the released chlorophenol red was measured at 590 nm using 650 nm as the reference wavelength as previously described (Zhang et al. 2017). The background absorbance value of co-cultured system containing B3Z CD8 + T hybridoma cells and BMDCs without OVA was subtracted in each assay.

### Statistical analysis

A minimum number of mice required to perform a feasible and reproducible statistical analysis were consulted by a professional statistician before the study. Results were presented as Mean ± SEM. Statistical significance was determined by one-way analysis of variation (ANOVA) and least-significant difference (LSD) method (SPSS 17.0, SPSS Inc., Chicago, IL, USA). *P* < 0.05 was considered as statistically significant.

## Results

### Arsenic inhibits autophagic flux in primary BMDCs

**Fig. 1:**
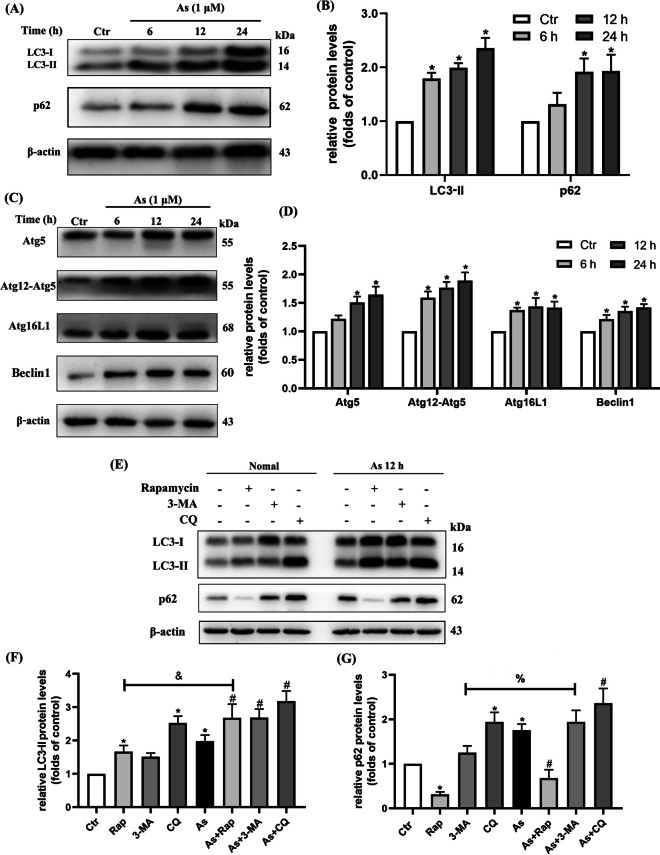
As inhibits autophagy flux in mouse primary BMDC cultures. **A** Western blotting of LC3-II and p62 in BMDCs exposed to 1 μM As for the times indicated. **B** Quantification of LC3-II and p62 protein levels. **C** Western blotting of ATGs and Beclin1 exposed to 1 μM As for times indicated. **D** Quantification of ATGs and Beclin1 protein levels. **E** BMDCs were pre-treated separately with ( +)/without ( −) autophagy agonist (200 nM, Rapamycin), autophagy inhibitors (1 mM 3-MA and 20 μM CQ) 1 h prior to 1 μM As exposure for 12 h, and representative bands of LC3-II and p62 proteins are shown. **F**, **G** Quantification of LC3-II and p62 protein levels. All summary data are normalized to control, and values reflect Mean ± SEM, ^*^P < 0.05 compared with control (Ctr) group, ^#^*P* < 0.05 compared with As group, ^&^*P* < 0.05 compared with Rap group, ^%^*P* < 0.05 compared with 3-MA group, *n* = 3

As to the evaluation of autophagy, autophagosome accumulation could be measured by transmission electron microscopy, GFP-LC3 fluorescence puncta, and LC3-II and p62/SQSTM1 (hereafter as p62) protein expression as well. As we observed with 1-μM As exposure at 6 h, 12 h, and 24 h in primary BMDCs, the expression of LC3-II and p62 protein levels was significantly increased, indicating an enhancement of autophagic activity as well as a possible impairment of autophagic degradation (Fig. 1A–B). In addition, we also observed that As exposure resulted in the expression of autophagosome-related proteins such as Atg5, Atg12-Atg5, Atg16L1, and Beclin1 (Fig. 1C–D).

It is essential to determine autophagic flux to accurately estimate autophagic activity. Normally, autophagic flux is inferred on the basis of LC3-II turnover measured by western blot in both the presence and absence of lysosomal degradation by bafilomycin A1 or CQ (Klionsky et al. 2021). Further determination of the altered autophagy flux process was performed in the presence of the autophagy inducer rapamycin, as well as the autophagy inhibitors CQ and 3-MA. As shown in the results, co-treatment of rapamycin with As resulted in a further increase in LC3-II levels compared to rapamycin alone. As CQ is a lysosomal inhibitor by raising pH, the difference in LC3-II levels between co-treatment of CQ with As and the As alone group was much smaller than the difference between CQ alone and the control group, indicating that As exposure resulted in both autophagosome production and insufficient degradation of LC3-II. Additionally, since 3-MA acts as a PI3K inhibitor to interfere with the autophagosome formation, exposure to As in the presence of 3-MA resulted in an additional increase in p62 protein turnover compared to 3-MA alone, also suggesting an abnormal accumulation of p62 as an autophagic substrate (Fig. 1E–G).

Collectively, these results suggest that As exposure may impair autophagic flux in primary BMDCs, leading to insufficient autophagosome degradation and subsequent accumulation of autophagic substrate protein.

### Arsenic impairs lysosomal degradative function in primary BMDCs

The autophagy process consists of three key steps: the formation of autophagosome, the fusion of the autophagosome with lysosome, and the degradation of autolysosomal contents by intracellular lysosome. It is worth emphasizing that the lysosomal digestive competence is a crucial part of the well-balanced autophagic flux process.

**Fig. 2:**
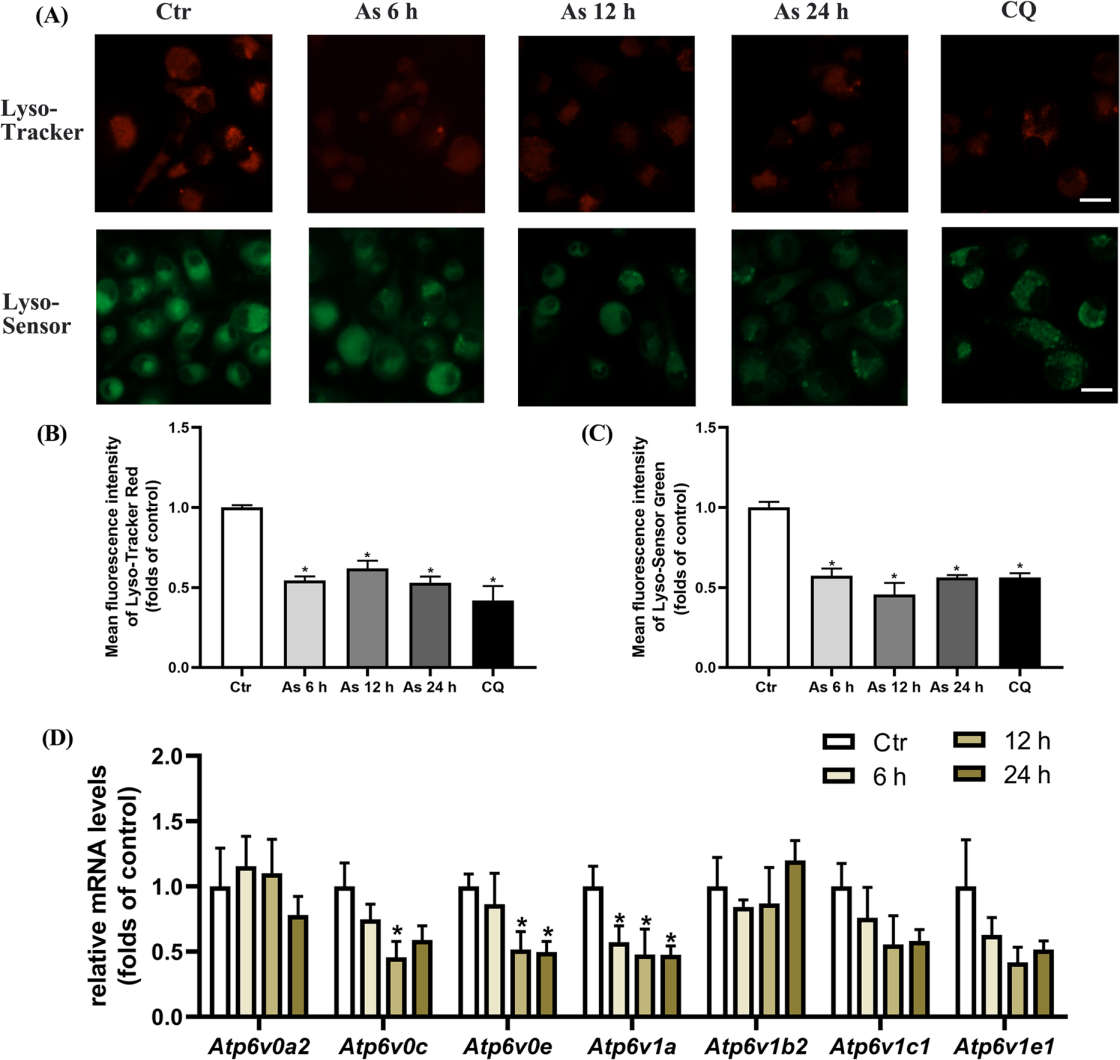
As impairs lysosomal degradative function in mouse primary BMDC cultures. **A** Immunofluorescence images stained with Lyso-Tracker Red or Lyso-Sensor Green treated with 1 μM As for the times indicated. **B**, **C** Quantification of Lyso-Tracker and Lyso-Sensor fluorescence intensity. Summary data from at least five visual fields in each independent experiment. **D** RT-PCR analysis showing mRNA expression of lysosome membrane proton pump genes exposed to 1 μM As for the times indicated. All summary data are normalized to control, and values reflect Mean ± SEM, scale bar: 20 μm, ^*^P < 0.05 compared with control (Ctr) group, *n* = 4

In our experiments, we then used Lyso-Tracker and Lyso-Sensor probes to assess lysosomal function. The results revealed that As exposure decreased the abundance of acidic lysosome and disrupted the acidic environment of the lysosome, with CQ as a positive control (Fig. 2A–C). Furthermore, the mRNA levels of *Atp6v0c*, *Atp6v0e*, and *Atp6v1a*, some of the key proton pump-related components that regulate lysosomal pH, were significantly reduced by As exposure in cultured BMDCs (Fig. 2D). Our results also showed that As exposure did not significantly block the fusion process of autophagosome with lysosome in cultured dendritic cells (Fig. S2), which prompted us to explore the possible impaired lysosomal degradative functions.

### Arsenic exposure leads to an increase in LMP of primary BMDCs

**Fig. 3:**
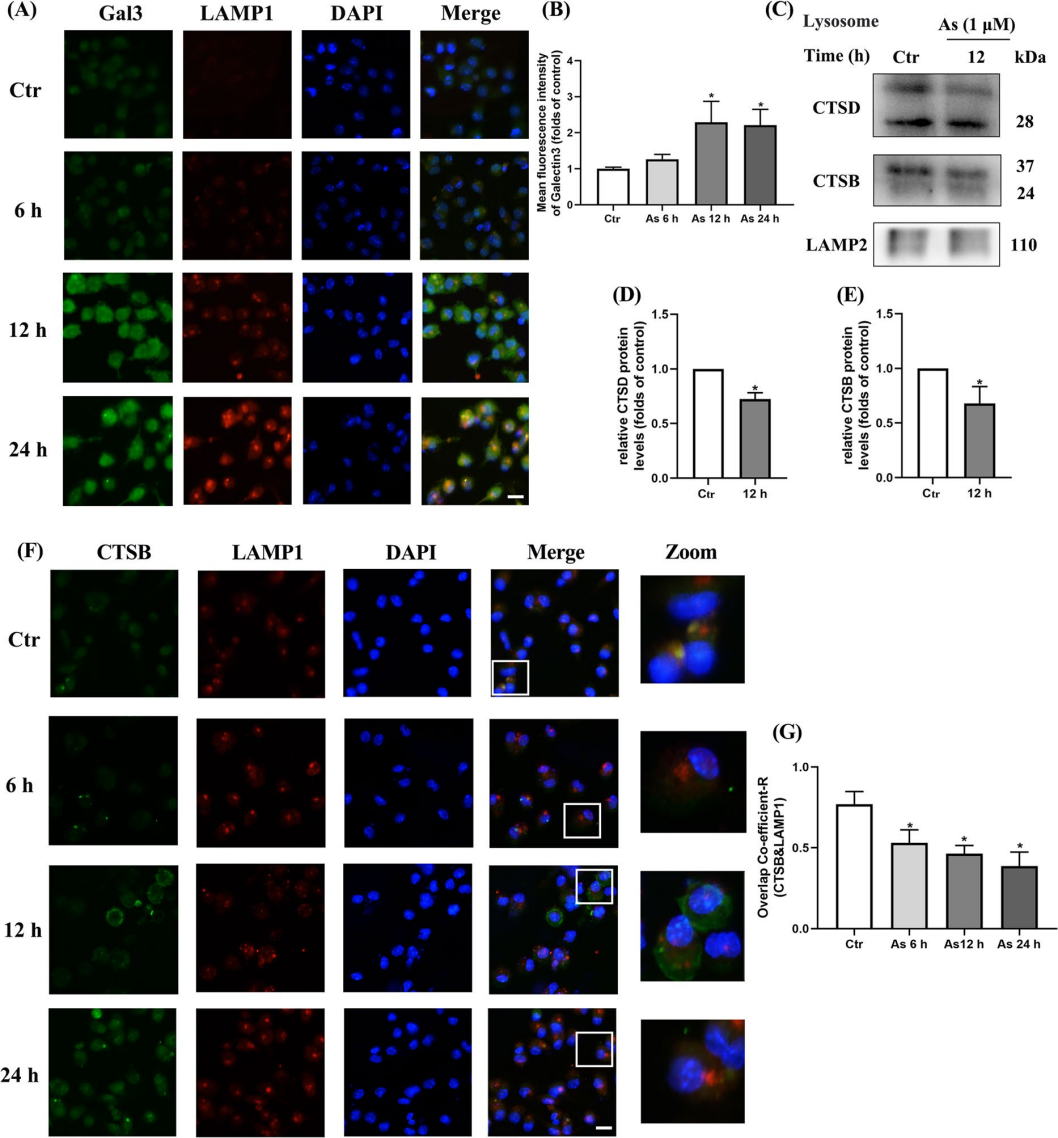
As exposure leads to increase LMP as well as altered lysosomal cathepsins levels and distribution in primary BMDCs. **A** Representative immunofluorescence images for Gal3 and LAMP1 treated with 1 μM As for the times indicated. **B** Quantitative analysis of Gal3 fluorescence intensity. **C** Lysosomal CTSD and CTSB proteins treated with 1 μM As for 12 h by western blotting. **D**, **E** Quantification of CTSD and CTSB protein levels. **F** Representative images show CTSB (green) immunostaining with LAMP1 (red) by 1 μM As for the times indicated. **G** Co-localization of **F** is represented by yellow signals and quantified as Pearson’s correlation coefficient. All summary data are normalized to control, and values reflect Mean ± SEM, scale bar: 20 μm, ^*^P < 0.05 compared with control (Ctr) group, *n* = 4

Lysosome is a membrane-enclosed organelle and the abnormal LMP results in the leakage of the intralysosomal components, which may also interfere with autophagic flux (Rusmini et al. 2019; Wang et al. 2018). Galactose lectin 3 (Gal3) is a lysosomal and endosomal membrane damage-specific marker that has been widely employed for investigating lysosomal membrane damage (Aits 2019). Immunofluorescence co-localization of Gal3 and LAMP1 (a lysosomal structural membrane protein) was employed in our experiments, and we found that Gal3 fluorescence gradually intensified, while the number of Gal3 fluorescent spots overlapping with LAMP1 gradually increased with prolonged exposure to As in primary BMDCs (Fig. 3A–B).

We further isolated lysosomes from primary BMDCs and detected the protein expression of intralysosomal cathepsins. The results showed a significant reduction in lysosomal CTSD and CTSB protein levels after exposure to As (Fig. 3C–E). Consistently, the distribution of lysosomal CTSB in primary BMDCs was also significantly reduced at 6 h, 12 h, and 24 h of As exposure, as evaluated by co-localisation of fluorescence-labeled CTSB and LAMP1 (Fig. 3F–G). Thus, our results suggest that an increase in LMP of primary BMDCs leads to leakage of lysosomal cathepsins and alters cathepsin distribution by As exposure.

### TFEB and TFE3 modulate arsenic-impaired lysosomal degradative function in BMDCs

As core regulatory transcription factors in the autophagy-lysosome pathway, TFEB and TFE3 have gained broad consensus for their essential role in a variety of human diseases and have become one of the hottest research topics in recent years (Sardiello et al. 2009). In this study, we next established both TFEB or TFE3 knockdown and overexpression models of BMDCs to further explore their potential effects on As-induced autophagic and lysosomal dysfunction, as described in the “Materials and methods” section (Fig. S3).

**Fig. 4:**
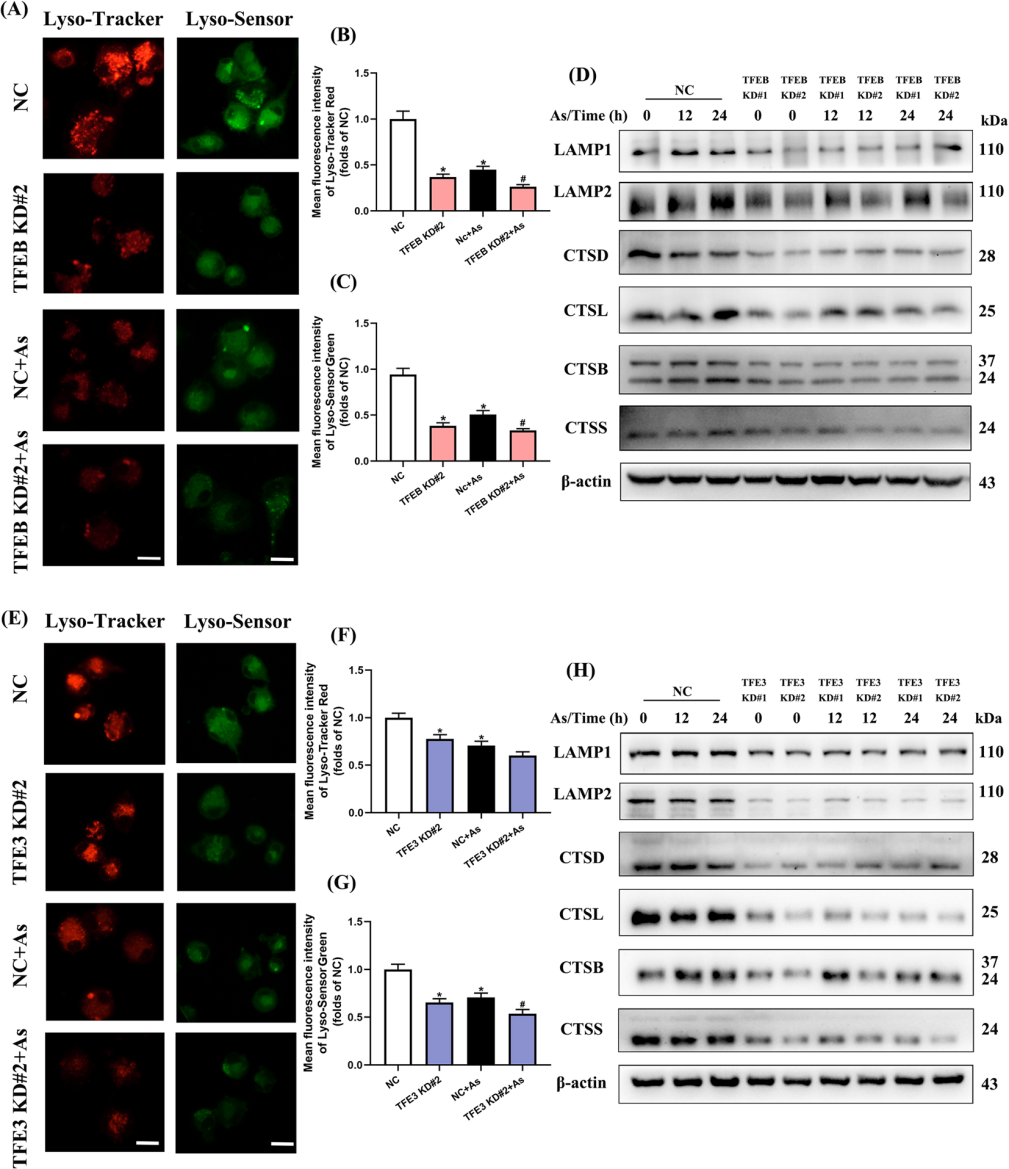
Knockdown of TFEB/TFE3 enhances lysosomal dysfunction in As-exposed primary BMDCs. Primary BMDCs were electro-transfected with plasmid carrying sgRNA-mediated knockdown of endogenous TFEB/TFE3 or NC plasmid. **A** Immunofluorescence images stained with Lyso-Tracker Red and Lyso-Sensor Green and exposed to 1 μM As for 12 h with TFEB knockdown. **B**, **C** Fluorescence intensity of Lyso-Tracker Red or Lyso-Sensor Green quantification, respectively (*n* = 4). **D** Western blotting of lysosome-associated proteins exposed to 1 μM As with TFEB knockdown for the times indicated. **E** Immunofluorescence images stained with Lyso-Tracker Red and Lyso-Sensor Green and exposed to 1 μM As for 12 h with TFE3 knockdown. **F**, **G** Fluorescence intensity of Lyso-Tracker Red or Lyso-Sensor Green quantification, respectively (*n* = 4). **H** Western blotting of lysosome-associated proteins exposed to 1 μM As with TFE3 knockdown for the times indicated. All summary data are normalized to negative control (NC), and values reflect Mean ± SEM, scale bar: 20 μm, ^*^P < 0.05 compared with NC group, ^#^*P* < 0.05 compared with NC + As group

**Fig. 5:**
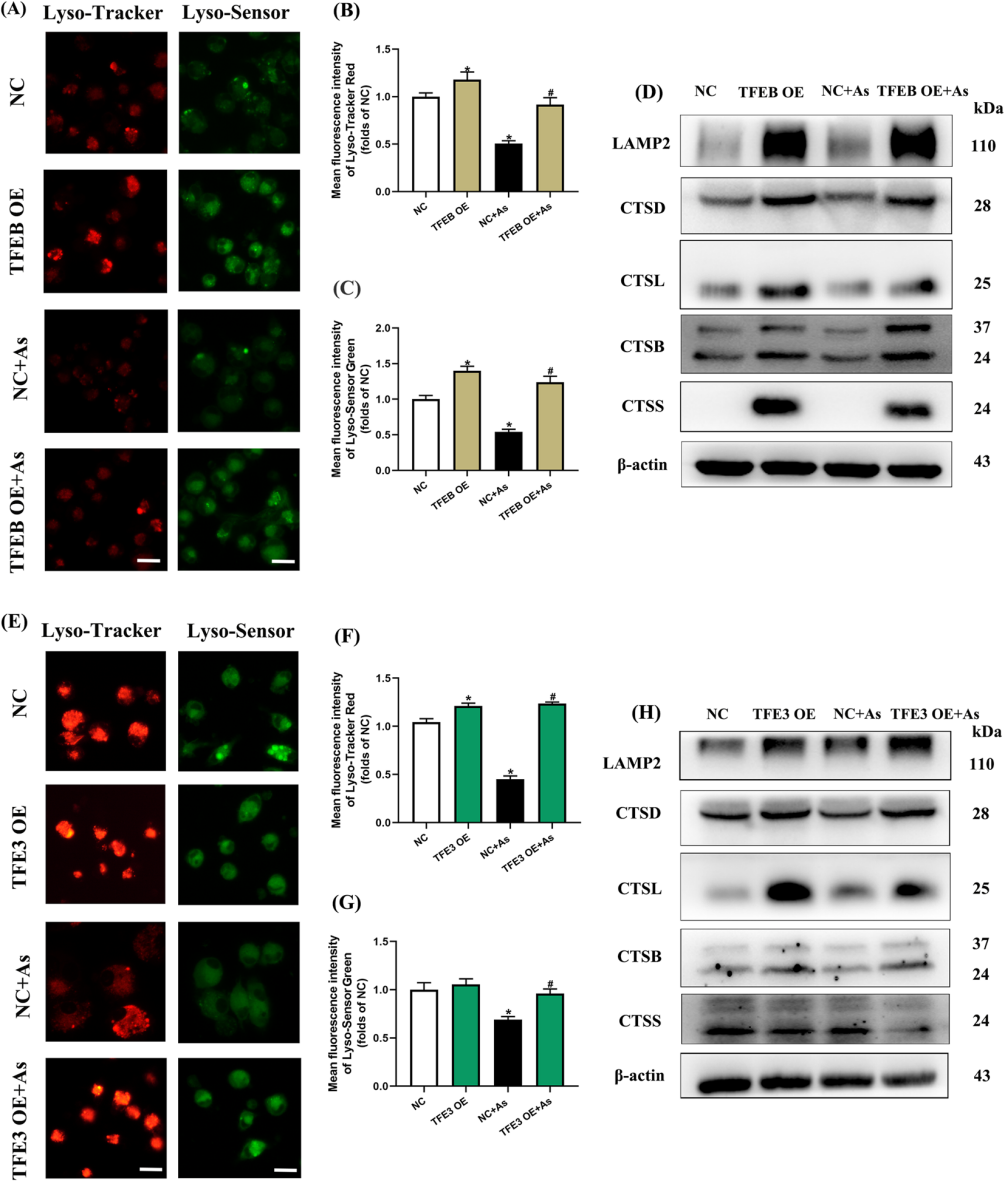
Overexpression of TFEB/TFE3 attenuates lysosomal dysfunction in arsenic-exposed primary BMDCs. Primary BMDCs were electro-transfected with plasmid carrying exogenous TFEB/TFE3 or empty vector and then exposed to 1 μM As for 12 h. **A** Immunofluorescence images stained with Lyso-Tracker Red or Lyso-Sensor Green and exposed to As with TFEB overexpression. **B**, **C** Fluorescence intensity of Lyso-Tracker Red or Lyso-Sensor Green quantification, respectively (*n* = 4). **D** Western blotting of lysosome-associated proteins exposed to As with TFEB overexpression. **E** Immunofluorescence images stained with Lyso-Tracker Red or Lyso-Sensor Green and exposed to As with TFE3 overexpression. **F**, **G** Fluorescence intensity of Lyso-Tracker Red or Lyso-Sensor Green quantification, respectively (*n* = 4). **H** Western blotting of lysosome-associated proteins exposed to As with TFE3 overexpression. All summary data are normalized to negative control (NC), and values reflect Mean ± SEM. Scale bar: 20 μm. ^*^P < 0.05 compared with NC group, ^#^*P* < 0.05 compared with NC + As group

We first observed that TFEB knockdown exacerbated the reduction in Lyso-Tracker and Lyso-Sensor fluorescence intensity (Fig. 4A–C), as well as the protein and mRNA expressions of lysosomal membrane proteins (LAMP1 and LAMP2) and lysosomal cathepsins (CTSD, CTSL, CTSB, and CTSS), compared with the corresponding As exposure groups (Fig. 4D, S4A). In contrast, the fluorescence intensity of Lyso-Tracker and Lyso-Sensor (Fig. 5A–C), as well as the expressions of lysosomal membrane proteins and cathepsins, was reversed apparently in TFEB overexpressing BMDCs (Fig. 5D, S4C).

We then observed that TFE3 knockdown exacerbated the reductions in Lyso-Tracker and Lyso-Sensor fluorescence intensity (Fig. 4E–G), lysosomal membrane protein, and cathepsin expressions compared with the corresponding As exposure groups (Fig. 4H, S4B), while TFE3 overexpression increased these lysosomal markers compared to As exposure alone (Fig. 5E–H, S4D).

**Fig. 6:**
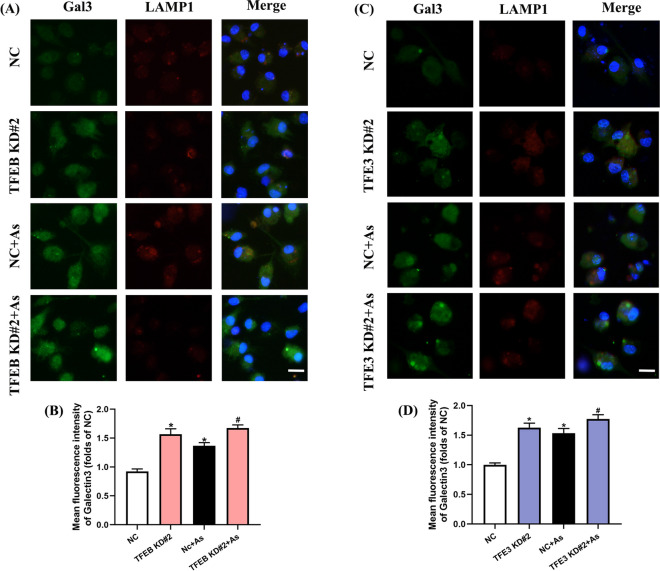
Knockdown of TFEB/TFE3 enhances LMP in As-exposed primary BMDCs. **A**, **C** Representative immunofluorescence images for Gal3 and LAMP1 exposed to 1 μM As for 12 h with TFEB or TFE3 knockdown, and quantitative analysis of the corresponding Gal3 fluorescence intensity are shown in **B** and **D**, respectively. All summary data are normalized to negative control (NC), and values reflect Mean ± SEM. Scale bar: 20 μm. ^*^P < 0.05 compared with NC group, ^#^*P* < 0.05 compared with NC + As group, *n* = 4

**Fig. 7:**
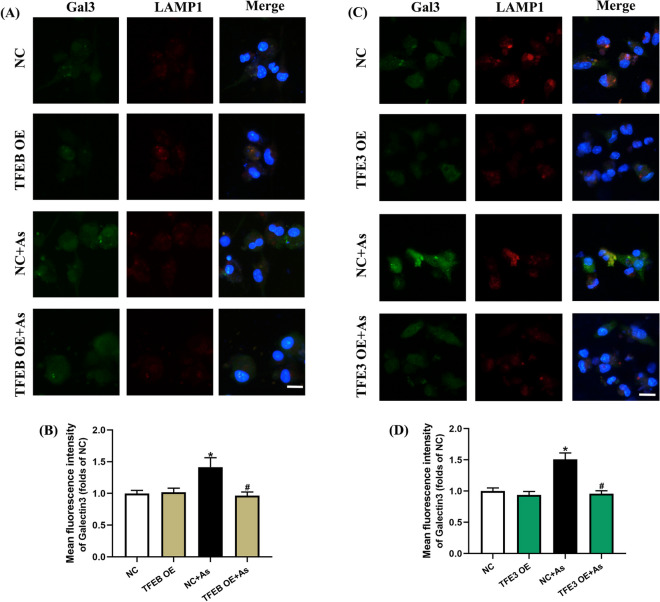
Overexpression of TFEB/TFE3 attenuates LMP in arsenic-exposed primary BMDCs. **A**, **C** Representative immunofluorescence images for Gal3 and LAMP1 exposed to 1 μM As for 12 h with TFEB or TFE3 overexpression, and quantitative analysis of the corresponding Gal3 fluorescence intensity are shown in **B** and **D**, respectively. All summary data are normalized to negative control (NC), and values reflect Mean ± SEM. Scale bar: 20 μm. ^*^P < 0.05 compared with NC group, ^#^*P* < 0.05 compared with NC + As group, *n* = 4

Consistent with these results, as indicators of lysosomal LMP, Gal3 fluorescence and co-localization of Gal3 and LAMP1 were also further enhanced in arsenic-treated cells with either TFEB or TFE3 knockdown (Fig. 6), while attenuated accordingly by both TFEB and TFE3 overexpression (Fig. 7).

### TFEB and TFE3 affect arsenic-inhibited autophagic flux in BMDCs

**Fig. 8:**
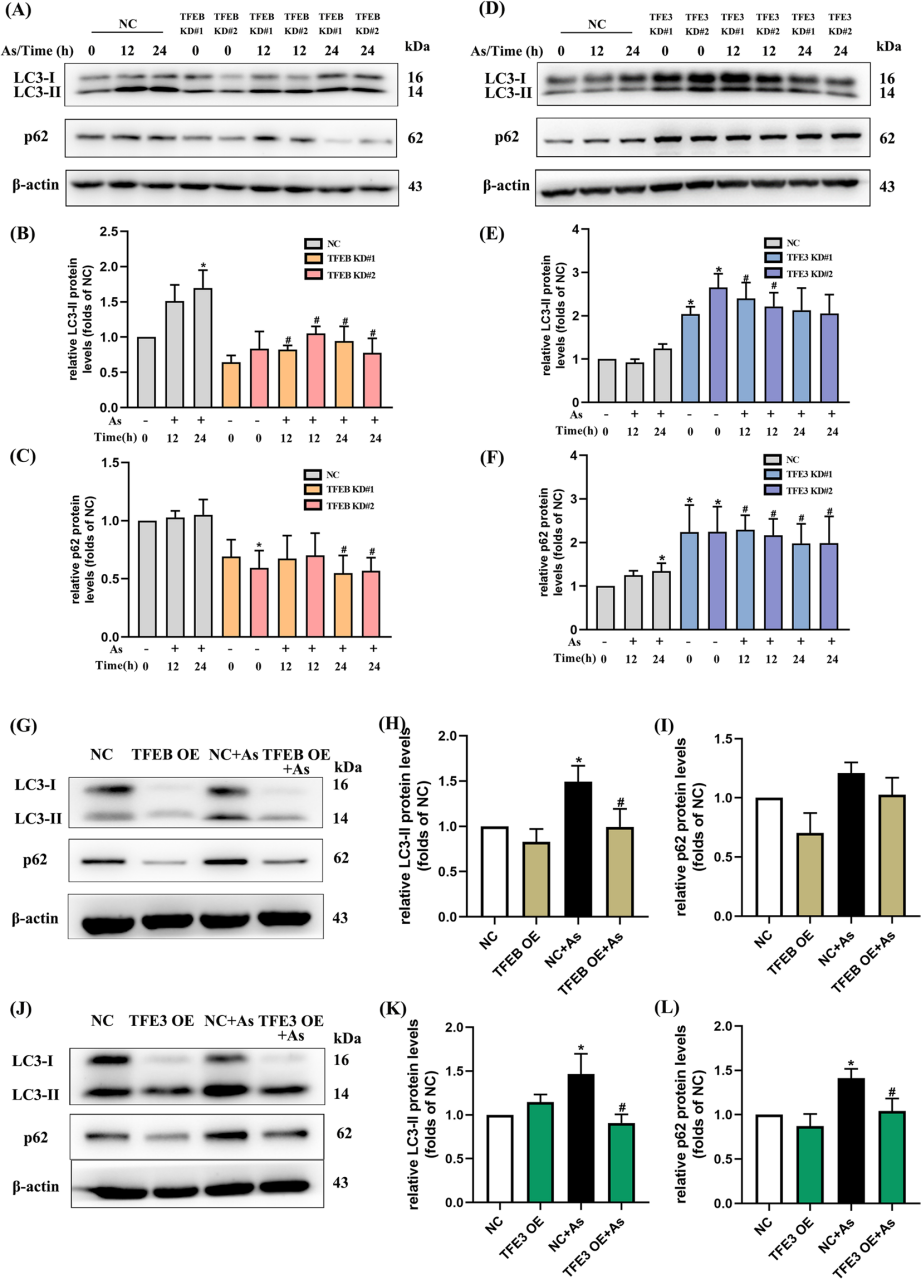
Autophagy flux is affected by knockdown or overexpression of TFEB/TFE3 in As-exposed primary BMDCs. **A**, **D** Western blotting of LC3-II and p62 exposed to 1 μM As with TFEB or TFE3 knockdown for the times indicated, quantitative analysis are shown in **B**–**C** and **E**–**F**, respectively (*n* = 3). **G**, **J** Western blotting of LC3-II and p62 exposed to 1 μM As with TFEB or TFE3 overexpression for 12 h, quantitative analysis are shown in **H**–**I** and **K**–**L**, respectively (*n* = 6). All summary data are normalized to negative control (NC), and values reflect Mean ± SEM. ^*^P < 0.05 compared with NC group, ^#^*P* < 0.05 compared with NC + As group

Regarding the effects of TFEB and TFE3 on As-induced autophagic dysfunction, our results indicated that the protein levels of both LC3-II and p62 were significantly decreased after TFEB downregulation, comparing with the corresponding arsenic alone group (Fig. 8A–C). Meanwhile, As-exposed groups with both TFEB and TFE3 overexpression exhibited a remarkable decrease in LC3-II and p62 proteins (Fig. 8G–L). However, we observed an increase in LC3-II and p62 protein expression by TFE3 knockdown, which is not yet very clear (Fig. 8D–F).

### TFEB and TFE3 regulate phenotypic molecules and cytokine expression in As-exposed BMDCs

**Fig. 9:**
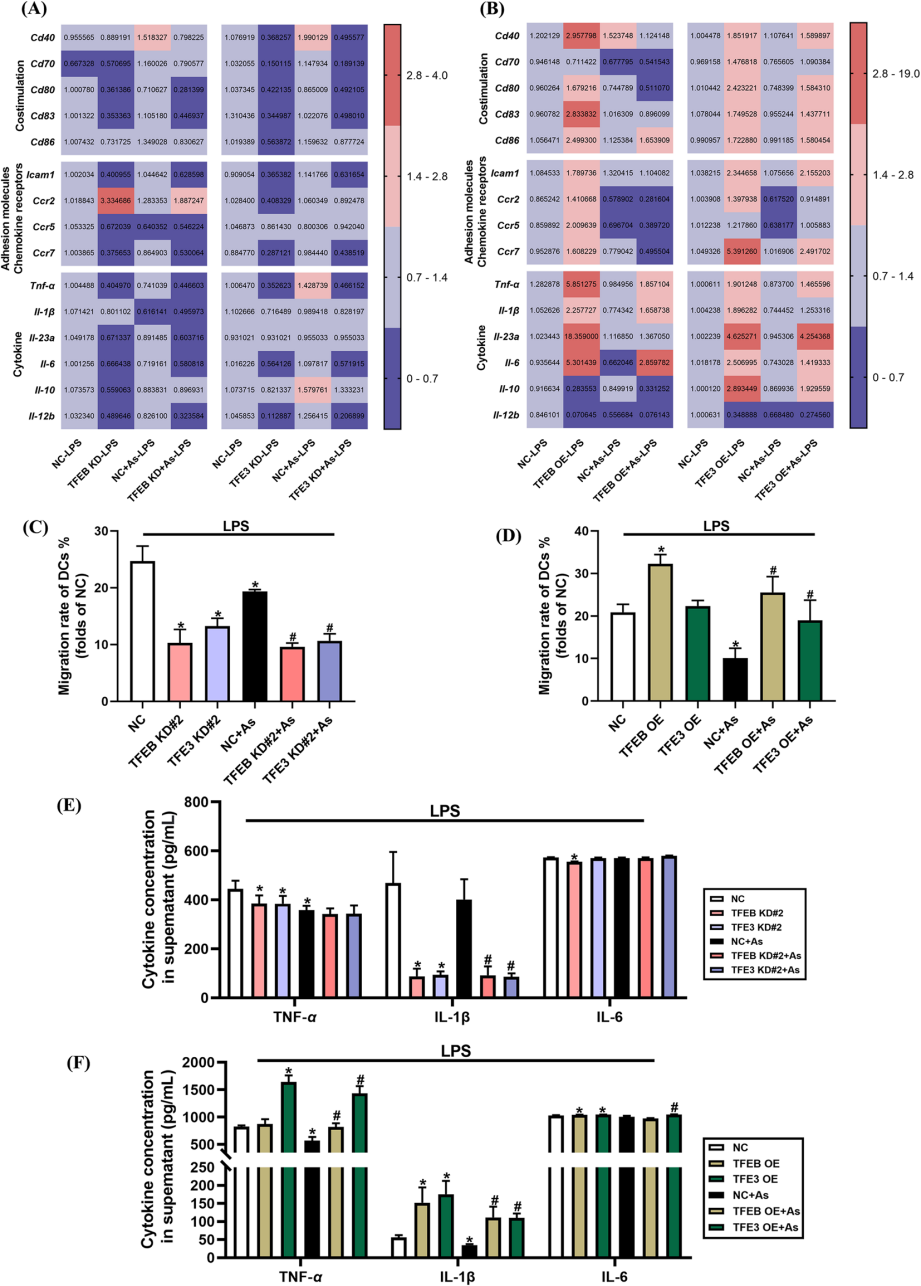
Knockdown or overexpression of TFEB/TFE3 on phenotypic molecules and cytokine expression in As-exposed primary BMDCs. **A**, **B** BMDCs were treated with 1 μM As for 2 h, stimulated with 25 ng/mL LPS for another 6 h with TFEB/TFE3 knockdown or overexpression. RT-PCR was performed and a heat map was generated to show the relative mRNA levels (Mean, *n* = 4) of nine phenotypic molecules and six cytokines, with significantly upregulated mRNA levels highlighted in red and significantly downregulated mRNA levels highlighted in purple. **C**–**F** BMDCs were treated with 1 μM As for 2 h, stimulated with 25 ng/mL LPS for another 12 h with TFEB/TFE3 knockdown or overexpression. Cell migration rate (**C**, **D**, *n* = 3) as well as TNF-α, IL-1β, and IL-6 secretion in the supernatants (**E**, **F**, *n* = 4) were determined by transwell migration and ELISA assay, respectively. All summary data are normalized to negative control (NC), and values reflect Mean ± SEM. ^*^P < 0.05 compared with NC group, ^#^*P* < 0.05 compared with NC + As group

RT-PCR was used to investigate the mRNA expression of co-stimulatory molecules, adhesion molecules, and chemokine receptors, as well as cytokines in LPS-treated BMDCs exposed to As. In our results, TFEB knockdown significantly reduced the expression of co-stimulatory molecules *Cd80* and *Cd83*, adhesion molecules, and chemokine receptors, as well as cytokines, compared to the corresponding As-exposed group. What’s more, the expression of co-stimulatory molecules, adhesion molecule *Icam1*, chemokine receptor *Ccr7*, and cytokines such as *Tnf-α*, *Il-6*, and *Il-12b* was significantly reduced by TFE3 knockdown (Fig. 9A). By contrast, overexpressing TFEB enhanced the mRNA expression of most cytokines, while the majority of the detected co-stimulatory molecules, adhesion molecules, and chemokine receptors, as well as cytokines, were all significantly reversed in TFE3-overexpressed BMDCs (Fig. 9B).

We further employed transwell migration assay to evaluate the migration rate of BMDCs. Our preliminary results have shown a significant reduction in the migration rate of DCs by As exposure (Fig. S5B), and in this paper, we further demonstrated that either TFEB or TFE3 knockdown exacerbated the reduction in migration rate, compared to the corresponding As exposure groups (Fig. 9C). Conversely, the migration rate could be reversed apparently in both TFEB- and TFE3-overexpressing BMDCs (Fig. 9D).

The levels of TNF-α, IL-1β, and IL-6 in the culture supernatant of BMDCs were further quantified by ELISA. Notably, IL-1β was significantly reduced in both TFEB- and TFE3-knockdown groups in contrast to the corresponding As exposure groups (Fig. 9E). Conversely, the levels of TNF-α and IL-1β were reversed by both TFEB and TFE3 overexpression (Fig. 9F).

### TFEB and TFE3 are involved in antigen-presenting function in As-exposed BMDCs

**Fig. 10:**
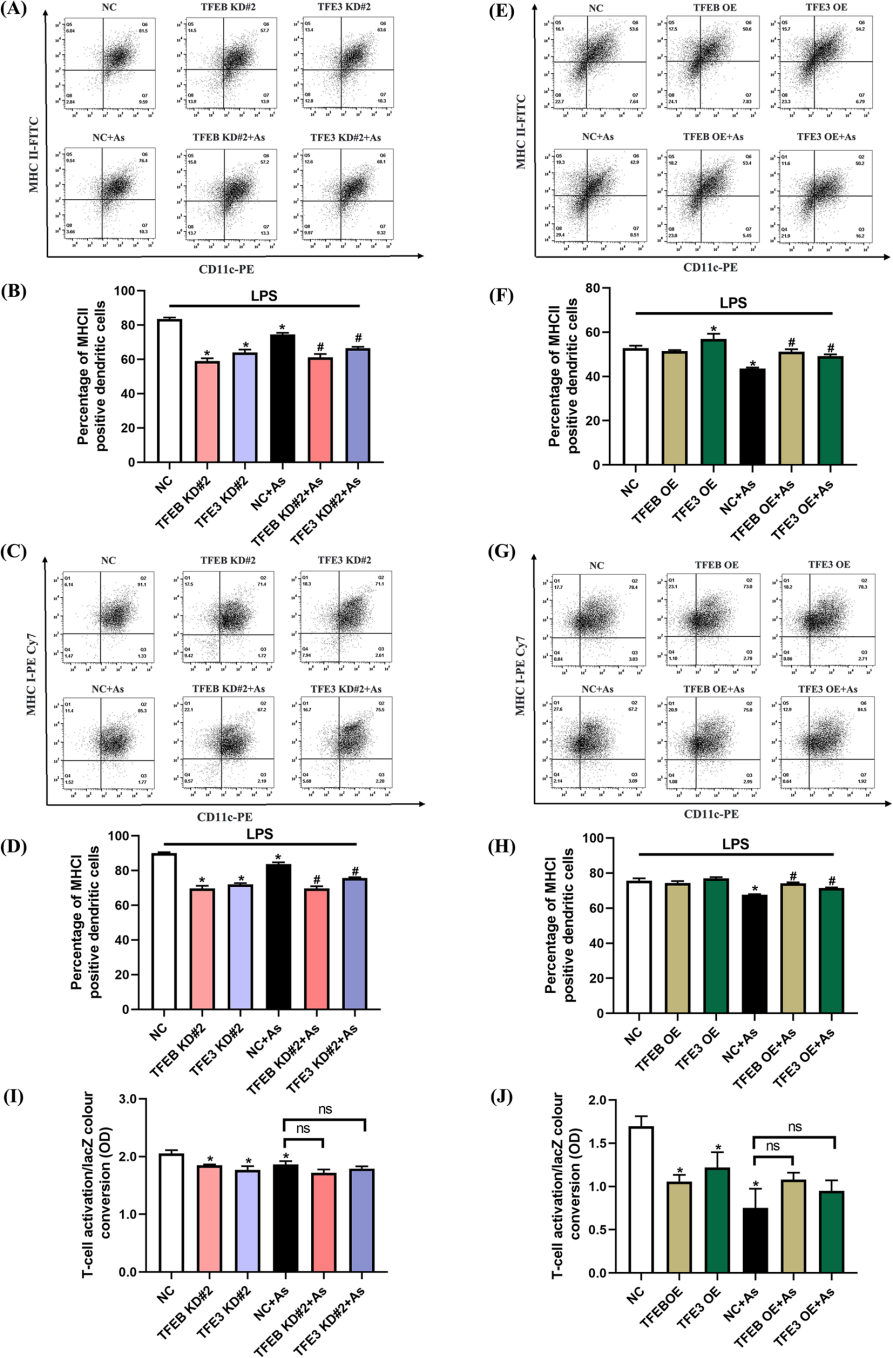
Knockdown or overexpression of TFEB/TFE3 on antigen presentation and antigen cross-presentation in As-exposed primary BMDCs. BMDCs were treated with 1 μM As for 2 h, stimulated with 25 ng/mL LPS for another 12 h with TFEB/TFE3 knockdown or overexpression. MHC II (**A**, **B**) and MHC I (**C**, **D**) molecules with TFEB or TFE3 knockdown, MHC II (**E**, **F**) and MHC I (**G**, **H**) molecules with TFEB or TFE3 overexpression were measured by flow cytometry and then quantified, respectively (*n* = 3). **I**, **J** Antigen cross-presentation was assessed using B3Z CD8 + T hybridoma cells co-cultured with BMDCs. BMDCs were treated with 1 μM As for 24 h with TFEB/TFE3 knockdown or overexpression, and activation of CD8 + T hybridoma cells was determined based on LacZ color conversion by optical density (OD) at 590 nm, *n* = 4. All summary data are normalized to negative control (NC), and values reflect Mean ± SEM. ^*^P < 0.05 compared with NC group, ^#^*P* < 0.05 compared with NC + As group

The two major classes of glycoproteins entrusted with antigen presentation are the MHC II and MHC I molecules, which present antigenic peptides to CD4 + T cells and CD8 + T cells, respectively (Pishesha et al. 2022). We next attempted to monitor changes in the double-positive molecules CD11c + MHC II + and CD11c + MHC I + on the surface of BMDCs to assess antigen-presenting function following by As exposure. Our results demonstrated that the expressions of MHC II and MHC I molecules were significantly reduced after TFEB or TFE3 was downregulated (Fig. 10A–D). Meanwhile, our results also confirmed that both TFEB and TFE3 overexpression increased the expressions of MHC II and MHC I molecules accordingly (Fig. 10E–H).

Cross-presentation refers to the presentation of exogenous antigens on MHC class I molecules, which is crucial for initiating cytotoxic immune responses (Segura and Amigorena 2015). In present study, we also explored the potential effect of As on cross-presentation function by co-culture of B3Z CD8 + T hybridoma cells with DCs. We have found that the secretion levels of IL-2 and IFN-γ in the co-culture supernatants, as well as the LacZ color conversion, were significantly reduced by As exposure (Fig. S6A–C); however, we did not observe the potential LacZ color conversion by either TFEB or TFE3 downregulation or upregulation (Fig. 10I–J).

## Discussion

**Fig. 11:**
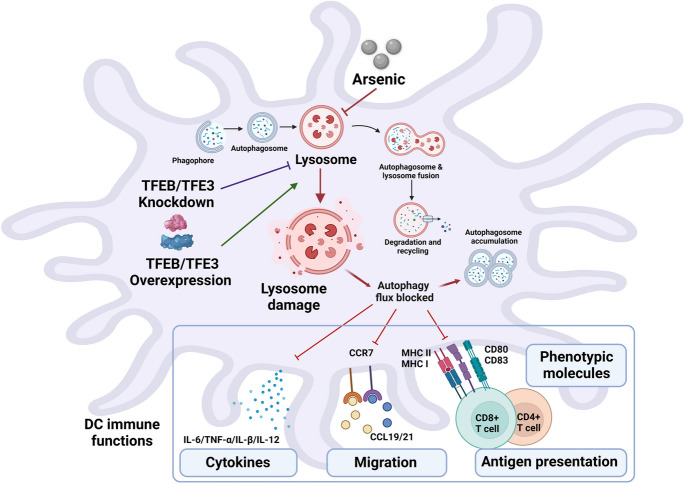
The proposed working model

In the present study, we investigated the mechanism underlying As-induced autophagic dysregulation in DCs and its functional role in As immunotoxicity. Our data suggest a potential role of lysosomal impairment in As-induced autophagy disorder and consequent aberrant immune response in DCs. We found that As exposure significantly impaired lysosomal number, lysosomal acidic environment, and LMP, resulting in blocked autophagic flux in DCs. Furthermore, as recognized transcriptional regulators of ALP, our results confirmed that TFEB or TFE3 knockdown exacerbated the impaired lysosomal degradative functions and the blockade of autophagic flux in As-exposed DCs. It also enhanced the inhibitory expression of co-stimulatory molecules *Cd80* and *Cd83*; adhesion molecule *Icam1*; cytokines TNF-α, IL-1β, and IL-6; chemokine receptor *Ccr7*; and antigen-presenting molecules MHC II and MHC I. By contrast, TFEB or TFE3 overexpression partially alleviated the As-induced inhibitory effects of DC lysosomal disorder, autophagic flux blockade, and immune dysfunction. The proposed working model is summarized in Fig. 11.

A growing number of studies have focused on the effects of As exposure on autophagy and autophagic flux, but the findings remain controversial. Studies have reported elevated expression of the autophagy-related proteins Atg-5, Beclin 1, and LC3-II, along with increased autophagic flux in Neuro-2a and human osteosarcoma cell lines, respectively (Fu et al. 2021; Wu et al. 2018). However, disrupted autophagosome-lysosome fusion and impaired autophagic flux with increased p62 protein levels have also been observed by acute arsenite exposure in cultured human keratinocytes (Wu et al. 2019). It is generally considered that reduced autophagosome formation, weakened autophagosome-lysosome fusion, and damaged lysosomal degradation could be the possible causes of impaired autophagic flux.

Accordingly, we first observed that As exposure impaired the autophagic flux process in cultured DCs, leading to insufficient degradation of the autophagosome protein LC3-II and accumulation of autophagic substrate protein p62, which was further verified by the autophagy inducer rapamycin as well as the autophagy inhibitors CQ and 3-MA. Our experiments then showed that As exposure did not significantly affect the fusion process of autophagosome with lysosome. However, As is also found to inhibit the autophagosome-lysosome fusion in NIH 3T3 cells by co-tranfected with the monomeric red fluorescent protein (mRFP)-GFP-LC3 fluorescent tandem reporter and LAMP1-CFP constructs (Dodson et al. 2018). Furthermore, as to the impaired lysosome-dependent degradation process, As-induced low levels of ROS could cause LMP and subsequent release of CTSB to the hepatic cytoplasm (Santra et al. 2022). Wang et al. have observed that trivalent arsenic exposure impairs lysosomal membrane stability in earthworms (Wang et al. 2016). In our results, we found that As apparently decreased the lysosomal abundance and disrupted the acidic environment of the lysosome by fluorescence probing. The mRNA levels of the key proton pump-related components that regulate lysosomal pH were also significantly reduced. In addition, increased LMP indicated by immunofluorescence co-localization of Gal3, a specific marker of lysosomal membrane damage, leakage of the lysosomal cathepsins CTSD and CTSB, as well as reduced distribution of CTSB on lysosomal membrane, were consistently confirmed in As-exposed DCs. Collectively, our series results systematically suggest that the impaired lysosomal degradation and membrane integrity of DCs is a possible mechanism by which As blocks autophagic flux and attenuates the degradation of autolysosomal contents in DCs.

TFEB and TFE3 are known to increase the number and activity of lysosomes by regulating the transcription of target genes involved in lysosomal biogenesis (Raben and Puertollano 2016). Overexpression of TFEB has shown protective effects on macrophages by inducing lysosomal genes (*Atp6v1h*, *Ctsd*, *Mcoln1*, and *Lamp1*), lysosomal compartment, and the number of lysosomes (Schilling et al. 2013; Zhang et al. 2022). TFE3 also induced macrophages LAMP1 and lysosomal abundance in a recent study (Pastore et al. 2016). In this paper, we next investigated the regulation of lysosomes and autophagy by TFEB and TFE3 in As-exposed DCs, respectively. Our results showed that TFEB or TFE3 knockdown significantly decreased the lysosomal abundance and disrupted its acidic environment. Meanwhile, the protein levels of the LAMP1, LAMP2, CTSD, CTSL, CTSB, and CTSS were also significantly reduced, consistent with increased LMP in As-exposed DCs. We further overexpressed TFEB or TFE3 of DCs by electrotransfection and found that the above-mentioned lysosomal disorders induced by As exposure could be partially alleviated. Consistent with our results, Fang et al. found that the fusion between autophagosomes and lysosomes, as well as lysosomal membrane protein LAMP1, was impaired in ATO-treated macrophages after TFEB knockdown (Fang et al. 2021). It is also worth mentioning that in our results, TFE3 showed a coordinated effect in restoring the number and function of lysosomes in As-exposed DCs.

It is suggested that restoration of autophagic flux could be achieved by improving lysosomal function through increased CTSD expression (Zhou et al. 2022). In motor neuron degeneration models, Rusmini et al. have reported that trehalose upregulated autophagy-related components (*Becn1*, *Atg12*, *Atg10*, *Map1lc3b*, *p62*, and *Hspb8*) in a TFEB-dependent manner (Rusmini et al. 2019). Consistent with these studies, our experiments here next demonstrated that TFEB knockdown downregulated the gene expression of *Map1lc3b* and *p62*, while TFEB or TFE3 overexpression showed a mitigating effect on As-induced autophagic flux blockade to some extent. Totally, our results here suggest that TFEB and TFE3 activating may contribute to lysosomal biogenesis and the expression of lysosome-associated genes in DCs, which in turn restores autophagic flux in As-exposed DCs.

Autophagy, a degradation process that occurs via the lysosomal pathway, has a critical role in regulation of innate and adaptive immune (Dong et al. 2021). We found that TFEB and TFE3 significantly modulated lysosomal dysfunction and autophagic flux blockade in DCs induced by As exposure. Therefore, the present study further investigated the possible modulatory role of TFEB and TFE3 in the suppression of immune functions (maturation, cytokine secretion, cell migration, and antigen presentation) of DCs by As exposure. The results of this study showed that TFEB or TFE3 knockdown enhanced the inhibitory expression of phenotypic molecules such as co-stimulatory molecules *Cd80* and *Cd83*; adhesion molecule *Icam1* and chemokine receptor *Ccr7*; migration rate; cytokines TNF-α, IL-1β, and IL-6; and antigen-presenting molecules MHC II and MHC I in As-exposed DCs. TFEB overexpression could alleviate the suppressive effect of As exposure on the above-mentioned immune functions. Impressively, TFE3 was likewise able to exert a protective effect in response to the suppressive effects of As exposure on DC immune functions.

The role of TFEB and TFE3 in regulating DC immune dysfunction caused by As exposure has not been reported. Although the modulatory effects of TFEB on DC functions have been documented, the results remain controversial due to the different microenvironments in which DCs reside. Contrary to the results of our study, Ding et al. demonstrated that knockdown of TFEB significantly enhanced the differentiation and maturation of tumor-educated DCs, as evidenced by upregulation of CD11c, CD86, and MHC II, while improving their ability to induce proliferation and differentiation of Th1 cells (Ding et al. 2022). On the other hand, Samie reported that TFEB expression in dendritic cells inhibited the presentation of exogenous antigens by MHC class I while enhancing antigen presentation by MHC class II (Samie and Cresswell 2015). In summary, our study confirmed that TFEB and TFE3 activated ALP and therefore may exert a collaboratively protective effect on As-induced immune dysfunction in DCs.

In addition to their ability to regulate the transcription of lysosomal biogenesis target genes, TFEB and TFE3 were found to regulate calcium release from lysosomes, which is required for rapid directional DC migration (Bretou et al. 2017). The present study observed that TFEB or TFE3 may regulate the migratory capacity of As-exposed DCs by altering the CCR7-CCL19/CCL21 axis. Furthermore, we did not observe a modulatory effect of TFEB or TFE3 on cross-antigen presentation in As-exposed DCs, although As impaired the cross-antigen presentation function of DCs. We have shown that either TFEB or TFE3 knockdown or overexpression significantly alters lysosomal degradative function and that mild pH and weak protein hydrolysis in lysosomes are important for cross-antigen delivery (Samie and Cresswell 2015). These require further in-depth study. In addition, post-translational modifications such as ubiquitination, phosphorylation, and acetylation may play a crucial character in the regulation of transcription factors such as TFEB and TFE3, whose nuclear shuttling is very challenging and deserves to be explored.

## Conclusions

Overall, our results suggest that As impairment of lysosomal degradative function in DCs is a likely mechanism by which As exposure blocks autophagic flux, further contributing to DC dysfunction. The key transcriptional regulators of the ALP, TFEB and TFE3 may contribute to lysosomal biogenesis and promote autophagy-related gene expression in DCs, thereby activating ALP and exerting a protective effect against As inhibition of immune function in DCs. We highlight TFEB and TFE3 as a potential therapeutic target to regulate the ALP and immune dysfunction in DCs caused by As exposure.

## Supplementary Information

Below is the link to the electronic supplementary material.

Supplementary file1 (DOCX 5449 KB)

## Data Availability

All data generated or analyzed during this study are included in this article. Additional raw data may be available from the corresponding author for reasonable reasons.

## Abbreviations

As: Arsenic
ALP: Autophagy-lysosome pathway
ATG: Autophagy-associated gene
BMDCs: Bone marrow-derived dendritic cells
CTSB: Cathepsin B
CTSD: Cathepsin D
CTSL: Cathepsin L
CTSS: Cathepsin S
CQ: Chloroquine
DCs: Dendritic cells
Gal3: Galactose lectin 3
LMP: Lysosomal membrane permeabilization
LAMP1: Lysosomal-associated membrane protein 1
LAMP2: Lysosomal-associated membrane protein 2
MHC II: Major histocompatibility complex II
MHC I: Major histocompatibility complex I
MAP1LC3/LC3: Microtubule-associated protein 1 light chain 3
OVA: Ovalbumin
SQSTM1/p62: Sequestosome 1
TFEB: Transcription factor EB
TFE3: Transcription factor E3
v-ATPase: Vacuolar-type H ^+^ -translocating adenosine triphosphatase
