# Supplementary material for: TFEB and TFE3 cooperate in regulating inorganic arsenic-induced autophagy-lysosome impairment and immuno-dysfunction in primary dendritic cells
Source: Cell Biol Toxicol. 2024 Jan 25;40(1):4. doi: 10.1007/s10565-024-09841-0 (PMC10808261; doi:10.1007/s10565-024-09841-0)
Supplement: Supplementary file 1 — Supplementary file1 (DOCX 5449 KB) [file 10565_2024_9841_MOESM1_ESM.docx]

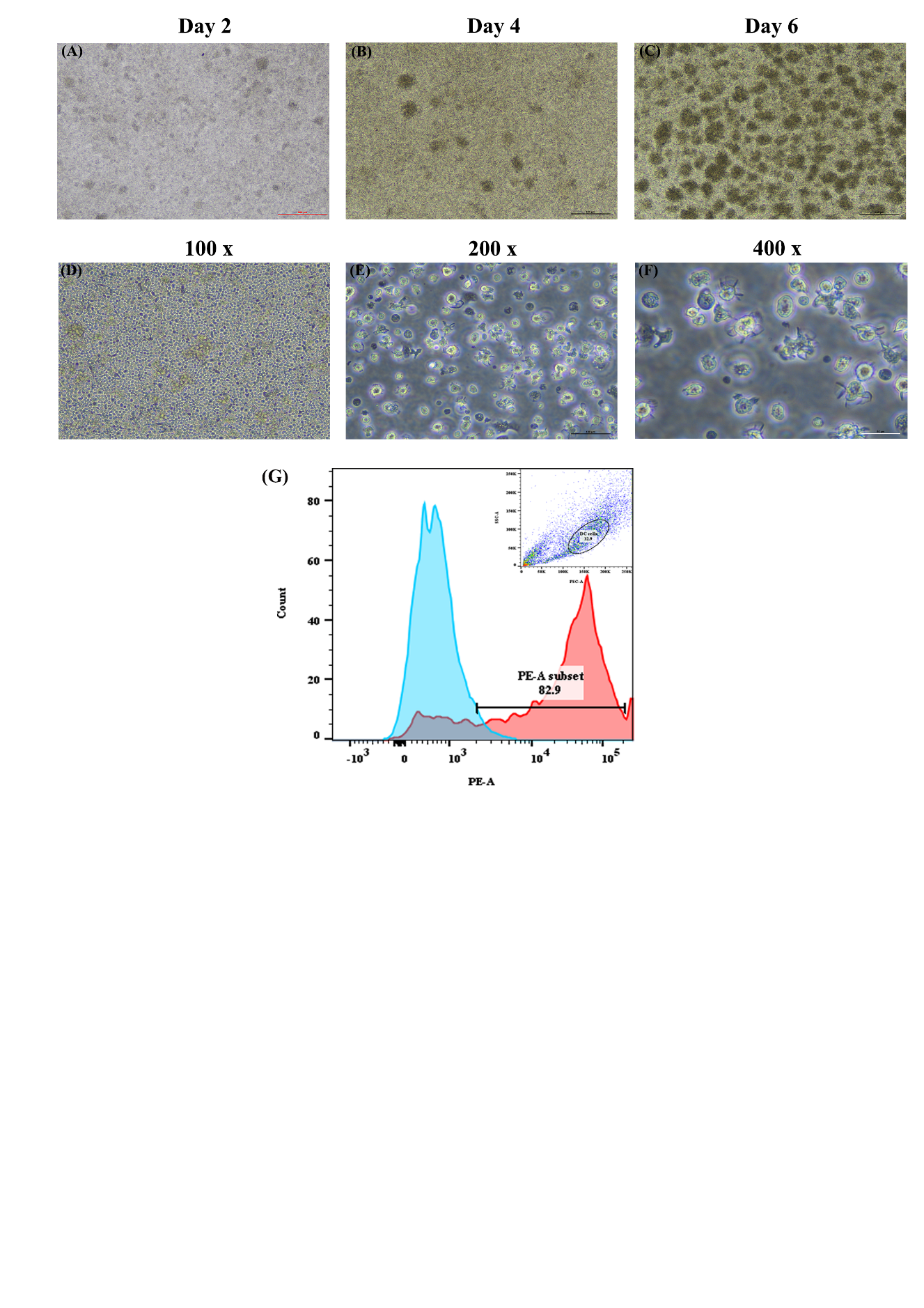


**Figure S1.** Primary cultured BMDCs are >80 % positive for PE-CD11c marker.


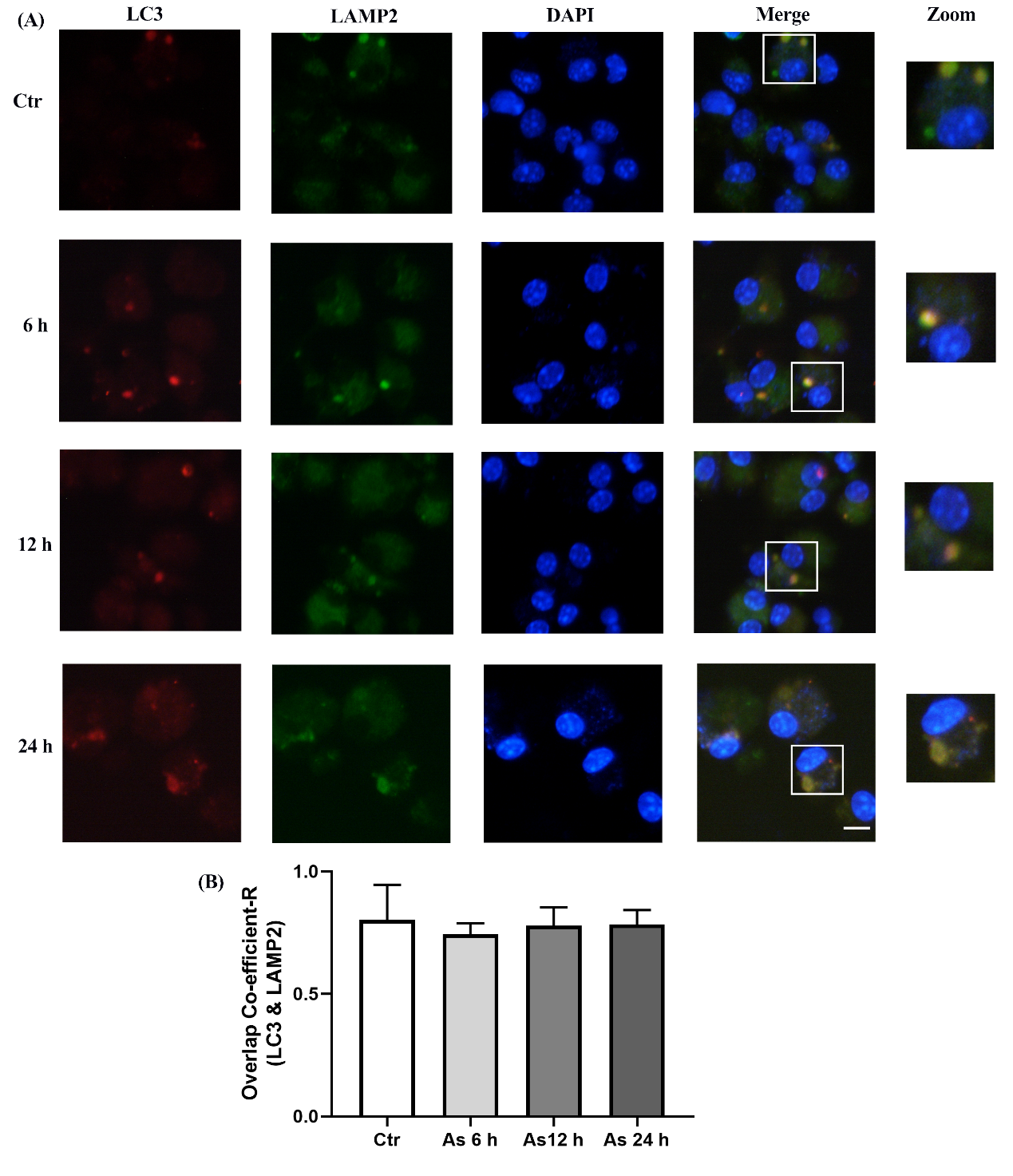


**Figure S2.** Effect of As exposure on autophagosome-lysosome fusion in primary BMDCs. (A) Representative images showing LC3 (red) immunostained together with LAMP2 (green). Co-localization of (A) is represented by yellow signals and quantified as Pearson’s correlation coefficient in (B), n=4. All summary data are normalized to control, and values reflect Mean ± SEM, Scale bar: 20 μm,


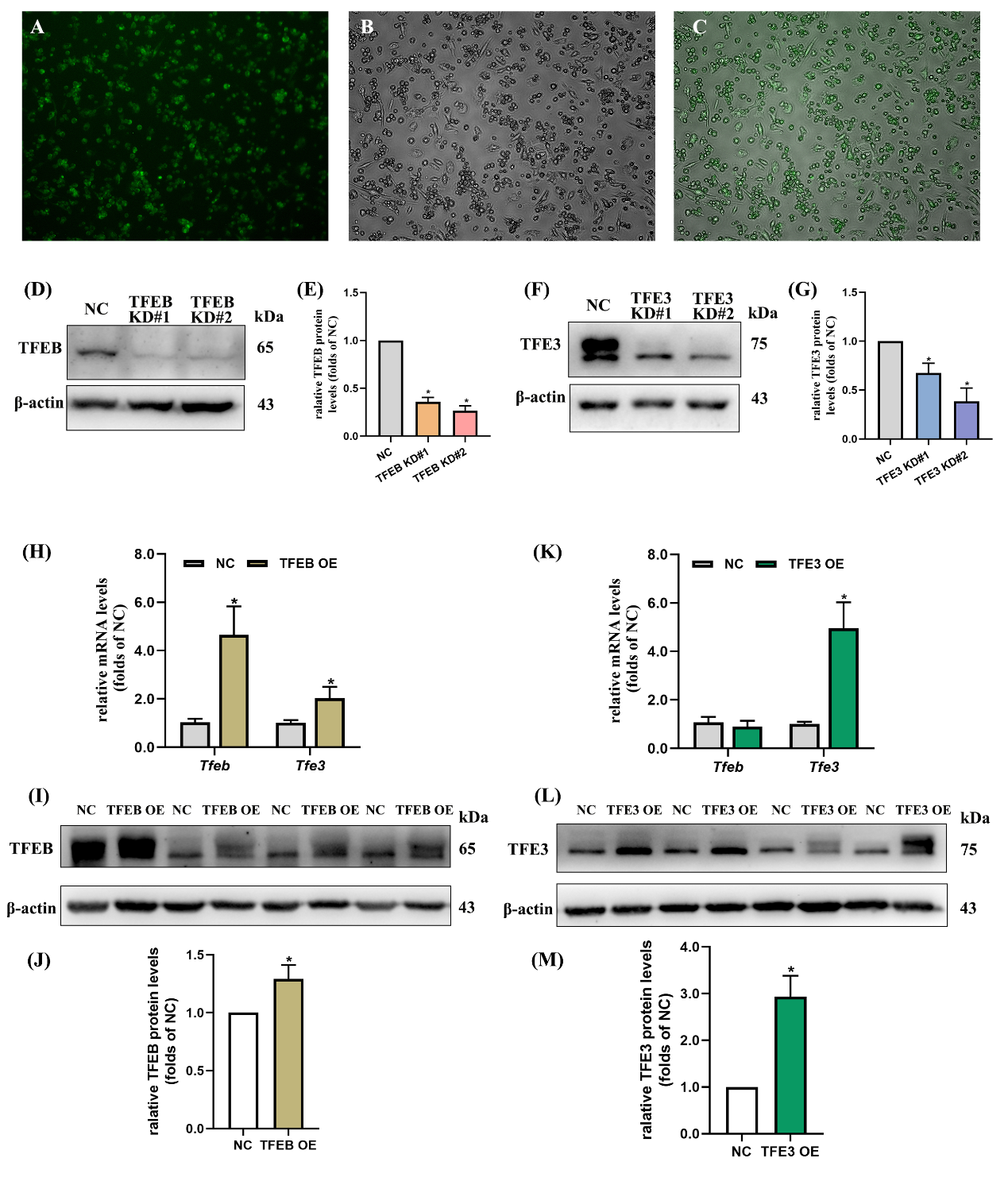


**Figure S3.** Knockdown or overexpression of TFEB/TFE3 in primary BMDCs is well established. (A-C) BMDCs were transfected with EGFP negative control plasmid. (D and F) Western blotting of TFEB and TFE3 proteins by TFEB/TFE3 knockdown in BMDCs. (E and G) Quantification of TFEB and TFE3 protein levels. (H and K) RT-PCR analysis showing mRNA expression of *Tfeb* and *Tfe3* genes by TFEB/TFE3 overexpression in BMDCs. (I and L) Western blotting of TFEB and TFE3 proteins by TFEB/TFE3 overexpression in BMDCs. (J and M) Quantification of TFEB and TFE3 protein levels. All summary data are normalized to negative control (NC), and values reflect Mean ± SEM. **^*^** *P*<0.05 compared with NC group, n=4.


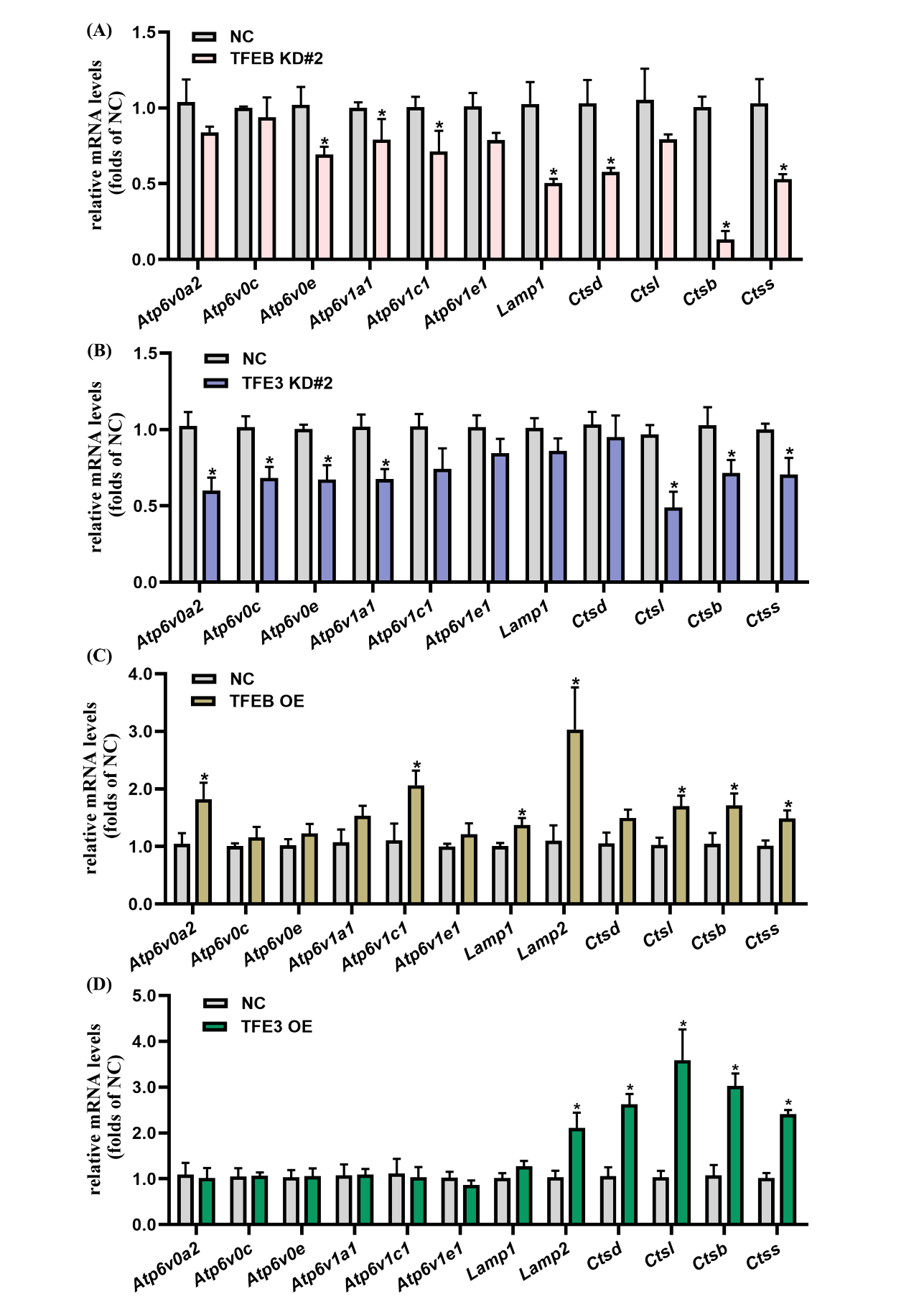


**Figure S4.** Knockdown or overexpression of TFEB/TFE3 regulates the expression of lysosome-related genes in primary BMDCs. RT-PCR results by TFEB/TFE3 knockdown (A-B) or overexpression (C-D) in BMDCs. All summary data are normalized to negative control (NC), and values reflect Mean ± SEM. **^*^** *P*<0.05 compared with NC group, n=4.


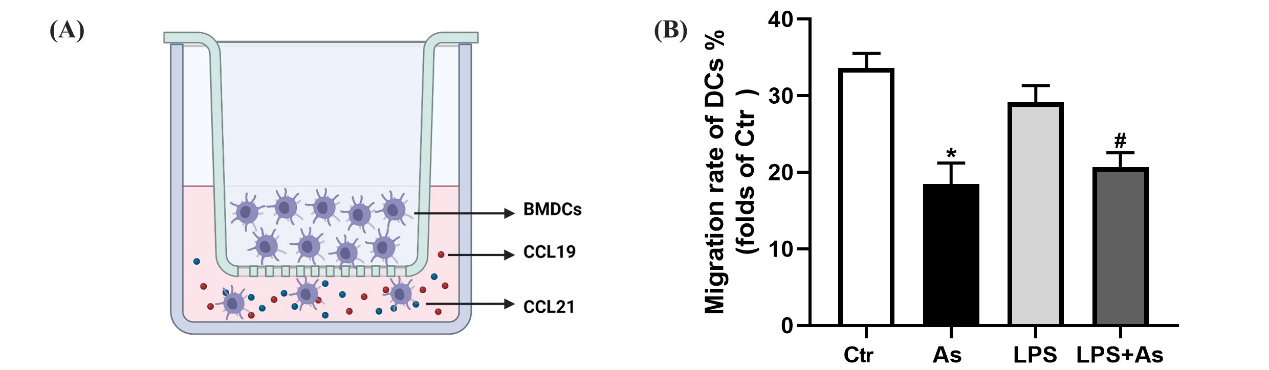


**Figure S5.** Effect of As exposure on migration rate in primary BMDCs. (A) Transwell migration experiment model diagram. (B) The changes of migration rate by arsenic exposure without or with LPS stimulation. All summary data are normalized to control (Ctr), and values reflect Mean ± SEM. **^*^** *P*<0.05 compared with Con group, **^#^** *P*<0.05 compared with LPS group, n=3.


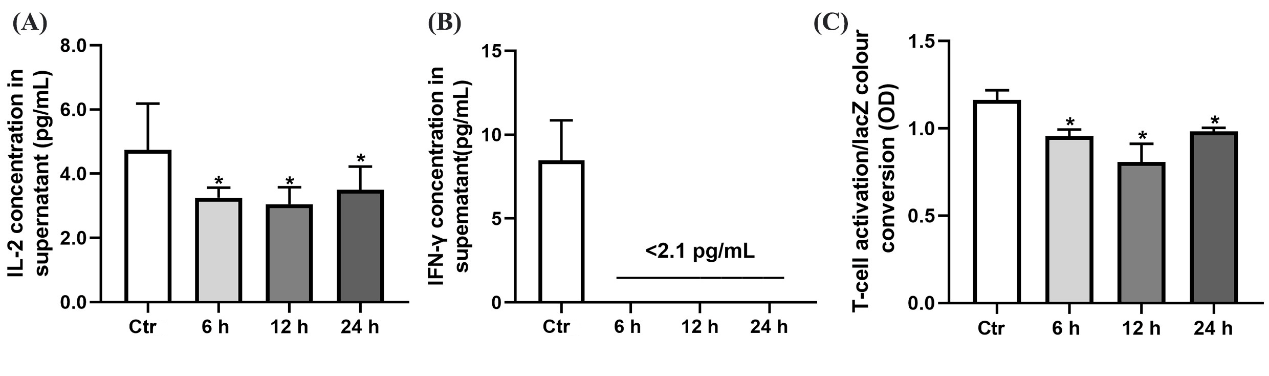


**Figure S6.** Effect of As exposure on antigen cross-presentation in primary BMDCs using B3Z CD8+T hybridoma cells coculture. Levels of (A) IL-2 and (B) IFN-γ in the coculture supernatant were determined by ELISA. (C) CD8+T hybridoma cell activation was determined based on LacZ colour conversion by optical density (OD) at 590 nm. All summary data are normalized to control (Ctr), and values reflect Mean ± SEM. **^*^** *P*<0.05 compared with Ctr group, n=4.

**Table S1.** Primary antibodies used in the western blot

| Antibody | Dilution | Catalog number | Supplier |
| --- | --- | --- | --- |
| LC3-II/LC3-I | 1：1000 | ab192890 | Abcam |
| p62/SQSTM1 | 1：1000 | ab56416 | Abcam |
| ATG5 | 1：1000 | D5F5U | Cell Signaling Technology |
| ATG12-ATG5 | 1：1000 | D88H11 | Cell Signaling Technology |
| ATG16L1 | 1：1000 | D6D5 | Cell Signaling Technology |
| BECLIN 1 | 1：1000 | A7353 | ABclonal |
| LAMP1 | 1：1000 | 55273-1-AP | Proteintech |
| LAMP2 | 1：1000 | ab25339 | Abcam |
| CTSD | 1：1000 | 21327-1-AP | Proteintech |
| CTSL | 1：500 | C-20：sc-390367 | Santa Cruz |
| CTSB | 1：1000 | ab58802 | Abcam |
| CTSS | 1：500 | C-20：sc-271619 | Santa Cruz |
| GAL3 | 1：1000 | 60207-1-lg | Proteintech |
| TFEB | 1：1000 | NBP2-41167 | Novus |
| TFE3 | 1：1000 | #14779 | Cell Signaling Technology |
| β-actin | 1：4000 | 20536-1-AP | Proteintech |

**Table S2.** Sequences of primers used in quantitative RT-PCR

| Gene (accession no.) | Primer sequence (5' to 3') | Product  size |
| --- | --- | --- |
| Mus-*Lamp1* | F:5'-TCTATGGCACTGCAACTGAA-3' | 147 bp |
| (NM_001317353.1) | R:5'-GGCTCTGTTCTTGTTCTCCA-3' |  |
| Mus- *Lamp2* | F:5'-GAGTGTTCGCTGGATGAT-3' | 229 bp |
| (NM_010685.4) | R:5'-GAGCAGGACTGTAACTAATGTA-3' |  |
| Mus-*Ctsd* | F:5'-AAGGTATCGCAGGGTGGAAA-3' | 193 bp |
| (NM_009983) | R:5'-CTGACAGTGGAGAAGGAGCA-3' |  |
| Mus-*Ctsb* | F:5'-CAGGCTGGACGCAACTTCTAC-3' | 104 bp |
| (NM_007798) | R:5'-TCACCGAACGCAACCCTTC-3' |  |
| Mus-*Ctsl* | F:5'-AGACCGGCAAACTGATCTCA-3' | 221 bp |
| (NM_009984) | R:5'-ATCCACGAACCCTGTGTCAT-3' |  |
| Mus-*Ctss* | F:5'-CATTGCCTGACACTGTGGAC-3' | 210 bp |
| (NM_006500975) | R:5'-CGCCTCCACAGCCTTTATTC-3' |  |
| Mus-*Atp6v0a2* | F:5'-TGGTGCAGTTCCGAGACCT-3' | 138 bp |
| (NM_011596.5) | R:5'-GCAGGGGAATATCAGCTCTGG-3' |  |
| Mus-*Atp6v0c* | F:5'-ACTTATCGCTAACTCCCTGACT-3' | 132 bp |
| (NM_001361531.1) | R:5'-ACACCAGCATCTCCGACGA-3' |  |
| Mus-*Atp6v0e* | F:5'-GCATACCACGGCCTTACTGT-3' | 109 bp |
| (NM_025272.2) | R:5'-TGATAACTCCCCGGTTAGGAC-3' |  |
| Mus-*Atp6v1a* | F:5'-ACAGAGGAAGCGTGACTTACA-3' | 110 bp |
| (NM_001358204.1) | R:5'-CACTTGGACCATGCTGAACTT-3' |  |
| Mus-*Atp6v1b2* | F:5'-ATGCGGGGAATCGTGAACG-3' | 114 bp |
| (NM_007509.3) | R:5'-AGGCTGGGATAGGTAGTTCCG-3' |  |
| Mus-*Atp6v1c1* | F:5'-ACTGAGTTCTGGCTCATATCTGC-3' | 106 bp |
| (NM_025494.3) | R:5'-TGGAAGAGACGGCAAGATTATTG-3' |  |
| Mus-*Atp6v1e1* | F:5'-GAATCAAGCAAGGCTCAAAGTCC-3' | 105 bp |
| (NM_007510.3) | R:5'-CGGGTCGTATCTTTTACCACC-3' |  |
| Mus-*Tfeb*  (NM_006524007) | F:5'-TGAGATGCAGATGCCTAACACGCT-3'  R:5'-TTGTCTTTCTTCTGCCGCTCCTTG-3' | 189 bp |
| Mus-*Tfe3* | F:5'-CGAGCCGTGTTCCTGCTATT-3' | 191 bp |
| (NM_001105196) | R:5'-TGCAGTGATATTGGGAGGCTG-3' |  |
| Mus-*Cd40*  ([NM_170702](http://www.ncbi.nlm.nih.gov/entrez/viewer.fcgi?db=nucleotide&id=70608176)) | F:5'-GCCATCGTGGAGGTACTGTT-3'  R:5'-CTGCGATGGTGTCTTTGCCT-3' | 110 bp |
| Mus-*Cd70* | F:5'-AGGAGGGCCATCTGCGTAT-3' | 199 bp |
| (NM_011617.2) | R:5'-AGGCGCTGTAATGCCACTG-3' |  |
| Mus-*Cd80*  (NM_009855) | F:5'-ACCCCCAACATAACTGAGTCT-3'  R:5'-TTCCAACCAAGAGAAGCGAGG-3' | 102 bp |
| Mus-*Cd83*  ([NM_009856](http://www.ncbi.nlm.nih.gov/entrez/viewer.fcgi?db=nucleotide&id=583966106)) | F:5'-CGCAGCTCTCCTATGCAGTG-3'  R:5'-GTGTTTTGGATCGTCAGGGAATA-3' | 136 bp |
| Mus-*Cd86*  ([NM_019388](http://www.ncbi.nlm.nih.gov/entrez/viewer.fcgi?db=nucleotide&id=161484598)) | F:5'-CTGGACTCTACGACTTCACAATG-3'  R:5'-AGTTGGCGATCACTGACAGTT-3' | 131 bp |
| Mus-*Icam1* | F:5'-TCCGCTACCATCACCGTGTAT-3' | 226 bp |
| (NM_010493.3) | R:5'-TAGCCAGCACCGTGAATGTG-3' |  |
| Mus-*Ccr2*  (NM_009915) | F:5'- ATCCACGGCATACTATCAACATC-3′  R:5'- CAAGGCTCACCATCATCGTAG-3′ | 104 bp |
| Mus-*Ccr5*  (NM_009917) | F:5'-TTTTCAAGGGTCAGTTCCGAC-3′  R:5'-GGAAGACCATCATGTTACCCAC-3′ | 158 bp |
| Mus-*Ccr7* | F:5'-TGTACGAGTCGGTGTGCTTC-3' | 162 bp |
| (NM_007719) | R:5'-GGTAGGTATCCGTCATGGTCTTG-3' |  |
| Mus-*Tnfα*  (NM_013693) | F:5'-CCTGTAGCCCACGTCGTAG-3'  R:5'-GGGAGTAGACAAGGTACAACCC-3' | 148 bp |
| Mus-*Il1β*  (NM_008361) | F:5'-TTCAGGCAGGCAGTATCACTC-3'  R:5'-GAAGGTCCACGGGAAAGACAC-3' | 75 bp |
| Mus-*Il12b*  ([NM_001303244](http://www.ncbi.nlm.nih.gov/entrez/viewer.fcgi?db=nucleotide&id=735997433)) | F:5'-TGGTTTGCCATCGTTTTGCTG-3'  R:5'-ACAGGTGAGGTTCACTGTTTCT-3' | 123 bp |
| Mus-*Il6*  (NM_001314054) | F:5'-TACCACTCCCAACAGACCTG-3'  R:5'-GGTACTCCAGAAGACCAGAGG-3' | 248 bp |
| Mus-*Il23a*  ([NM_031252](http://www.ncbi.nlm.nih.gov/entrez/viewer.fcgi?db=nucleotide&id=133892789)) | F:5'-TATCCAGTGTGAAGATGGTTGTG-3'  R:5'-CACTAAGGGCTCAGTCAGAGTTG-3' | 367 bp |
| Mus-*Il10*  (NM_010548) | F:5'-CTTACTGACTGGCATGAGGATCA-3'  R:5'-GCAGCTCTAGGAGCATGTGG-3' | 101 bp |
| Mus-*Gapdh*  (NM_001289726) | F:5'-TGTGTCCGTCGTGGATCTGA-3'  R:5'-TTGCTGTTGAAGTCGCAGGAG-3' | 150 bp |

**Table S3.** Primary antibodies used in the immunofluorescence assay

| Antibody | Dilution | Catalog number | Supplier |
| --- | --- | --- | --- |
| LC3 | 1：100 | ab192890 | Abcam |
| LAMP1 | 1：100 | 55273-1-AP | Proteintech |
| LAMP2 | 1：100 | ab25339 | Abcam |
| CTSB | 1：100 | ab58802 | Abcam |
| GAL3 | 1：100 | 60207-1-lg | Proteintech |

**Table S4.** Gene sgRNA target sequences

| Gene | Primer sequence (5' to 3') |
| --- | --- |
| *Tfeb* sgRNA1（mouse） | GCTGCCATGGCGTCACGCAT |
| *Tfeb* sgRNA2（mouse） | CCTCTGTGGATTACATCCGG |
| *Tfe3* sgRNA1（mouse） | AATGGTGAGCAGCGCCATG |
| *Tfe3* sgRNA2（mouse） | CGTGTAGGGTTCTCGAGGT |
| NC sgRNA | GACGCTTAACGACTGTTATG |
